# Zygosity-based sex determination in a butterfly drives hypervariability of *Masculinizer*

**DOI:** 10.1126/sciadv.adj6979

**Published:** 2024-05-03

**Authors:** Arjen E. van’t Hof, Ivy Whiteford, Carl J. Yung, Atsuo Yoshido, Magda Zrzavá, Maaike A. de Jong, Kian-Long Tan, Dantong Zhu, Antónia Monteiro, Paul M. Brakefield, František Marec, Ilik J. Saccheri

**Affiliations:** ^1^ Department of Evolution, Ecology and Behaviour, University of Liverpool, Liverpool L69 7ZB, UK.; ^2^ Biology Centre of the Czech Academy of Sciences, Institute of Entomology, 370 05 České Budějovice, Czech Republic.; ^3^ Faculty of Science, University of South Bohemia, 370 05 České Budějovice, Czech Republic.; ^4^ Netherlands eScience Center, Science Park 402, 1098 XH Amsterdam, Netherlands.; ^5^ Department of Biological Sciences, National University of Singapore, Singapore 117543, Singapore.; ^6^ Department of Zoology, University of Cambridge, Cambridge CB2 3EJ, UK.

## Abstract

Nature has devised many ways of producing males and females. Here, we report on a previously undescribed mechanism for Lepidoptera that functions without a female-specific gene. The number of alleles or allele heterozygosity in a single Z-linked gene (*BaMasc*) is the primary sex-determining switch in *Bicyclus anynana* butterflies. Embryos carrying a single *BaMasc* allele develop into WZ (or Z0) females, those carrying two distinct alleles develop into ZZ males, while (ZZ) homozygotes initiate female development, have mismatched dosage compensation, and die as embryos. Consequently, selection against homozygotes has favored the evolution of spectacular allelic diversity: 205 different coding sequences of *BaMasc* were detected in a sample of 246 females. The structural similarity of a hypervariable region (HVR) in *BaMasc* to the HVR in *Apis mellifera csd* suggests molecular convergence between deeply diverged insect lineages. Our discovery of this primary switch highlights the fascinating diversity of sex-determining mechanisms and underlying evolutionary drivers.

## Teaser

Male-killing cost of a primary sex determination switch maintains genetic diversity through heterozygote advantage.

## INTRODUCTION

Comparison of sex determination systems across animal lineages has revealed a pattern of high diversity and evolutionary turnover of the primary signal that initiates female or male development (*1*, *2*). In insects, the identity of these primary signals, and how they function, has been described in only a handful of species (*3*–*9*). For instance, in the model lepidopteran *Bombyx mori*, sex is determined by WZ (female) and ZZ (male) sex chromosomes, where the presence of the W chromosome–linked *Feminizer* (*Fem*) locus generates *Fem* Piwi-interacting RNA, essential for female development, and down-regulates a Z-chromosome–linked gene (*Masculinizer*), essential for male development (*5*). Here, we report the discovery of a primary sex determination mechanism in the Afrotropical butterfly *Bicyclus anynana* that differs fundamentally from the mechanism found in *B. mori*. This is unexpected because both species share the common WZ/ZZ sex chromosome system of Lepidoptera (*10*).

Our study was initially stimulated by the detection of a recessive lethal genomic interval on the Z chromosome containing *B. anynana Masculinizer* (*BaMasc*), which manifested as a deficit of homozygotes (males) in the adult progeny of daughter-father backcrosses (*11*). To test the role of *BaMasc* in this lethality, we excluded the genes other than *BaMasc* within the lethal interval and performed experiments to determine the association and timing of death during development among three categories of *BaMasc* genotypes, grouped by zygosity (i.e., hemizygous, heterozygous, or homozygous). We investigated the underlying cause of *BaMasc* homozygote embryonic death via associations with sex-specific splicing of *B. anynana doublesex* (*Badsx*), showing that ZZ *BaMasc* heterozygotes produce the male *Badsx* isoform, WZ *BaMasc* hemizygotes produce the female *Badsx* isoform, but ZZ *BaMasc* homozygotes also produce the female isoform of *Badsx* and fail to dosage compensate. We confirmed the causal link with *BaMasc* by demonstrating, experimentally, that *BaMasc* knockdown leads to feminization of *Badsx*. To identify the regions within *BaMasc* that control this mechanism, we characterized the distinctive structural features and sequence polymorphism of *BaMasc* and went on to describe the evolutionary consequences of heterozygote advantage for the haplotype diversity of this region in a wild-caught sample from across Africa. Last, through the detection of reproductively competent females lacking a W chromosome, we confirmed that this sex determination system does not require a sex-specific locus.

## RESULTS

### The homozygous lethal Z chromosome segment

The boundaries of the homozygous lethal region on the Z chromosome were not precisely defined in our previous study (*11*). On the basis of the position of homozygous genotypes in the mapping family reported in that study, the homozygous lethal interval spans ~1.6 Mb (from *scabrous* to *6-Pgd*). However, the backcross families that were produced for that study imply that the causal locus lies within a narrower region (*C5197* to *6-Pgd*). To define the interval more explicitly, we used recombination mapping in one of these backcross families (2BF_1_). A coarse mapping approach, using polymerase chain reaction (PCR)–based markers (table S1), on both parents and all 24 sons, produced an interval of 1.4 Mb (from “scaffold 2021” to *scabrous*), containing 33 genes (table S1). This interval was substantially reduced, by locating the closest recombination breakpoints via whole genome sequencing of targeted individuals in this family, to 482 kb (*Srebp* intron 7 to *CaaT* intron 1), containing 16 genes, including *BaMasc* (table S1).

**Fig. 1.**
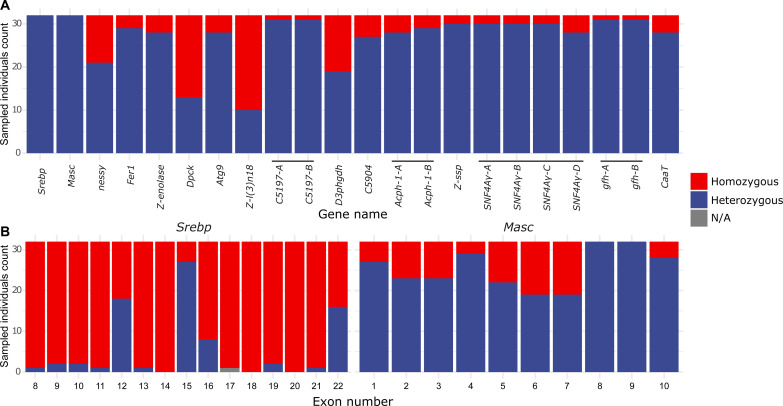
Pattern of genic homozygosity within the lethal interval. (**A**) The number of adult *B. anynana* males out of a sample of 32 (randomly sampled from partially inbred laboratory populations) that were completely homozygous at the amino acid level for the 16 genes (and their isoforms, distinguished by letters) present within the Z chromosome interval that was found to be homozygous lethal in backcross families. (**B**) For the two genes that did not have any coding sequence homozygotes at the level of the whole gene (*Srebp* and *Masc*), the distribution of coding sequence homozygosity at the exon level shows that the only exons for which homozygotes are completely absent in the sample (i.e., they are always heterozygous) are *Masc* exons 8 and 9 (*Srebp* exons 1 to 7 were excluded from the lethal interval by recombination mapping). N/A indicates individuals for which sufficient data were not available.

To examine the hypothesis that, within this 16-gene interval, the homozygous lethal effect was most strongly associated with *BaMasc*, as compared to any of the other genes, we sequenced whole genomes of 32 adult male individuals taken from captive Liverpool stock populations. Prolonged and episodically strong genetic drift and inbreeding since the establishment of the original laboratory population in 1988 (*11*), combined with decoupling of *BaMasc* genotype from flanking sequences through recombination, were expected to produce some homozygotes in genes that were not homozygous-lethal. At the gene level, we found that 14 of the 16 genes had at least one individual that was homozygous at the amino acid level [we did not consider introns, untranslated regions (UTRs), or intergenic regions]; the two genes remaining were *BaMasc* and its neighbor *Srebp* (Fig. 1A). At the exon level (Fig. 1B and fig. S1), *BaMasc* shows a consistently lower fraction of amino acid homozygotes across its 10 exons, with two exons (8 and 9) standing out for having no homozygotes. In contrast, *Srebp* does not show a pattern of high heterozygosity across all exons, and none of its exons are heterozygous in all individuals. *BaMasc* is a single copy gene in *B. anynana*, with the exception of pseudogenized copies on the W chromosome (*W-BaMasc*) which lack exons 6 to 10 (table S2).

### *B. anynana Masc* homozygotes die as embryos

We tested the hypothesis that *BaMasc* homozygotes die as embryos by investigating cosegregation between *BaMasc* genotype and lethality in the “Liverpool” laboratory population, descended from a wild sample collected in Malawi in 1988 (*11*). If *BaMasc* homozygosity is lethal, then half the male progeny from any mating where the parents share a *BaMasc* allele are expected to be inviable. Such progenies were obtained by collecting eggs from sister-brother matings screened for *BaMasc* genotype (fig. S2). Egg batches were incubated for 120 hours post-oviposition (hpo) and given ample opportunity to hatch (at 26°C, viable embryos hatch after a maximum of 108 hpo; movie S1), before all eggs and larvae were frozen. Virtually all of the unhatched eggs exhibited a specific “black egg” phenotype, which comes from the darkened head capsule visible through the eggshell, starting at 78 to 90 hpo (movie S1). Representative samples of unhatched eggs and larvae from each of 21 full-sibling crosses (*n* = 29 to 112 per family) were sex-karyotyped using a molecular WZ/ZZ assay (fig. S3) and genotyped for a variable region of *BaMasc* exon 9 (table S3).

**Fig. 2.**
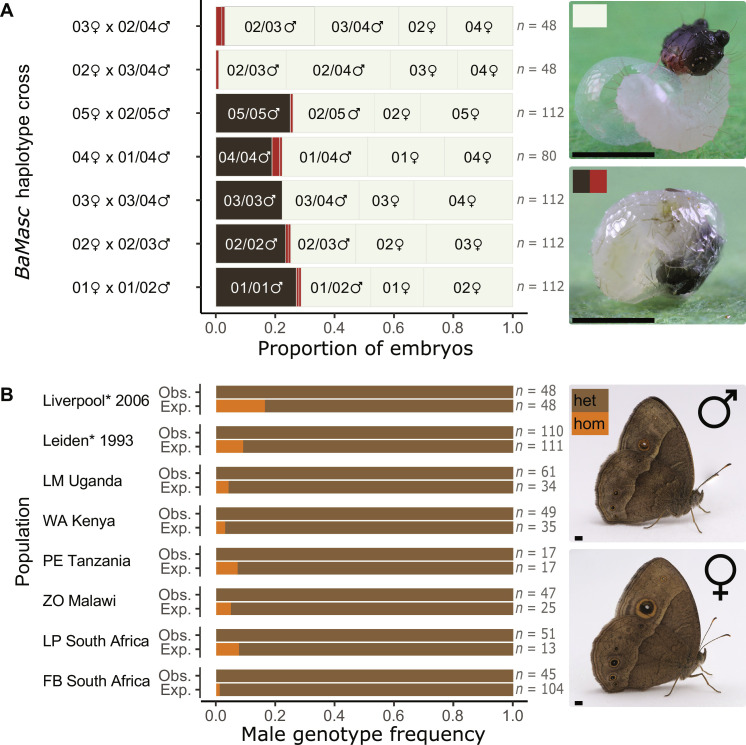
Lethality of *BaMasc* homozygotes. (**A**) The proportions of different categories of viable (light gray) and inviable (black or maroon) embryos produced by seven sister-brother crosses. The crosses differ in their complement of *BaMasc* haplotypes (indicated by haplotype numbers 01 to 05). No *BaMasc* homozygotes were possible in the two crosses where the parents did not share a *BaMasc* allele. The minor internal divisions within the nonhatching category (maroon shading) indicate embryos that were heterozygous or hemizygous for *BaMasc* (i.e., not homozygous). Images show a hatching larva (top) and a larva that has failed to hatch (bottom, black egg phenotype). Scale bars, 1 mm. (**B**) Observed (Obs.) proportions of *BaMasc* homozygotes (hom/orange): heterozygotes (het/brown) in adult male samples (of size *n*) from two related laboratory populations (*) and six wild samples collected in 2006–2008. These are compared to their expected (Exp.) frequencies based on contemporary female samples (of size *n*) from the same populations. Images of adult *B. anynana* male and female. Scale bars, 1 mm. Collection site abbreviations: LM, Lake Mburo; WA, Watamu; PE, Pemba; ZO, Zomba; LP, Limpopo; FB, False Bay (*31*).

The unhatched black eggs were karyotypically (ZZ) male and homozygous for *BaMasc*, with very few exceptions (Fig. 2A). Moreover, no *BaMasc* homozygotes were detected in the hatched larval sample (*n* = 611; table S4). This pattern held across all five *BaMasc* haplotypes present in the Liverpool population (*n* = 175 to 224 for each *BaMasc* haplotype). In contrast, black eggs were rare (1 to 3%) in families where the parents did not share a *BaMasc* haplotype, despite being related to the same degree (sister-brother). The proportions of karyotypic (ZZ) males to (WZ) females, *BaMasc* homozygotes to heterozygotes, and each of the paternal *BaMasc* haplotypes within daughters did not deviate significantly from Mendelian ratios in any of the families.

Further, if all *BaMasc* haplotypes are recessive lethals, then we should see no *BaMasc* homozygotes in adult populations, both in captivity and in the wild. This is indeed the case, in two related laboratory populations (Liverpool 2006 and its parental population Leiden 1993) and in wild-caught males sampled from geographically distinct locations in Africa (Fig. 2B). These populations showed deficit of homozygous males expected under Hardy-Weinberg equilibrium, with haplotype frequencies estimated from contemporary female samples. The deficit was most evident in the two laboratory populations (χ^2^: *P* < 0.001), which had fewer *BaMasc* alleles and therefore higher expected numbers of homozygotes (table S5).

### *BaMasc* homozygotes are feminized and die due to dysregulated dosage compensation

The black egg phenotype is characteristic of male-killing endosymbiont infections (*12*, *13*), often associated with improper dosage compensation (DC) of Z-linked genes due to interference with the sex determination pathway (*14*, *15*). In insects, this pathway uses a conserved switch, the sex-specific splicing of *doublesex* (*dsx*), downstream of a diverse array of primary signals, including *Masc* (*16*). Early disruption of *Masc* function in ZZ (normally male) embryos of *B. mori* and *Ostrinia furnacalis* moths results in expression of the female rather than male *dsx* splicing patterns (*5*, *17*). Given the central role of *Masc* in lepidopteran sex determination and the similarity of the lethal phenotype, we hypothesized that homozygosity of *BaMasc* was analogous to endosymbiont disruption, producing a mismatch between karyotypic (male) and genetic (female) sex, which subsequently interact atypically during the establishment of DC, resulting in developmental breakdown and nonviable embryos.

**Fig. 3.**
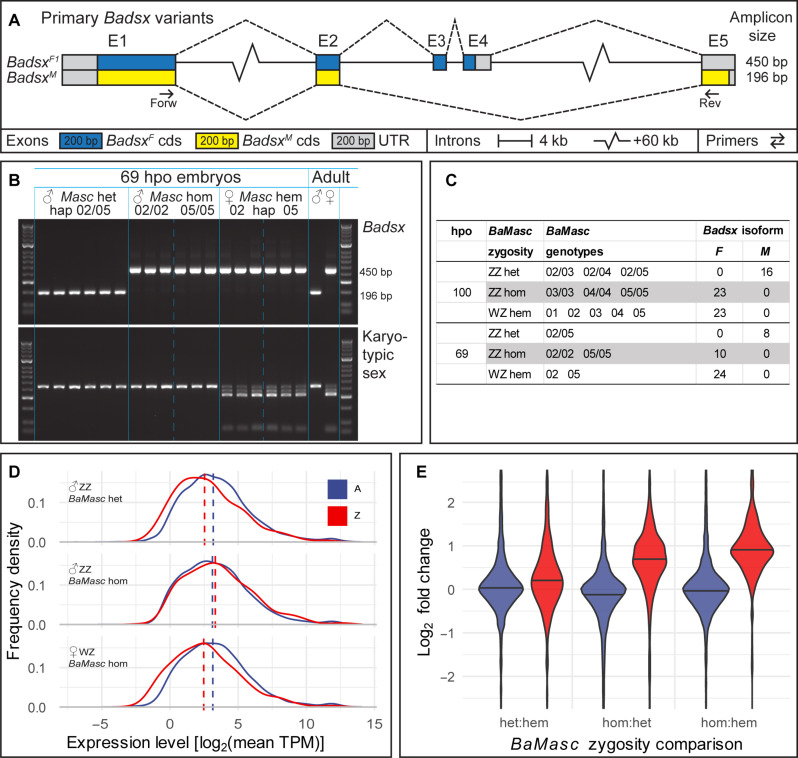
Mismatched karyotypic versus transcriptional sex leads to atypical DC. (**A**) Female (*Badsx^F^*) and male (*Badsx^M^*) splice variants of *Badsx*, with reference to the genomic intron-exon structure. Dashed lines connect exons (E1 to E5) in each transcript. (**B**) *Badsx* splice variants (top gel: 196-bp *Badsx^M^* or 450-bp *Badsx^F^*) in relation to *BaMasc* zygosity (heterozygotes, homozygotes, and hemizygotes for haplotypes 02 and 05) and karyotypic sex based on W-*BaMasc*/Z-*BaMasc* polymorphisms (bottom gel: ZZ single band or WZ four bands) in single 69 hpo embryos. Adults illustrate the characteristic patterns for males and females. (**C**) Summary of observations on *Badsx* isoform categories (female and male) in *B. anynana* embryos in relation to their *BaMasc* zygosity and genotype (specified by haplotype numbers). The absence of haplotype 01 in homozygous and heterozygous genotypes was due to its low frequency in the Liverpool stock during experiments. (**D**) Distributions of gene expression levels at 69 hpo of autosomal genes (blue) and Z-linked genes (red) in *BaMasc* heterozygotes, homozygotes, and hemizygotes [only genes with transcripts per million (TPM) > 1 in at least three samples are included: *n*_A_ = 9507, *n*_Z_ = 361]. Dashed lines indicate the median gene expression. Each distribution is based on RNA-seq of the same embryos shown in (B): three ZZ *BaMasc* heterozygotes (all *Masc*-02/05 genotype), six ZZ *BaMasc* homozygotes (three *BaMasc*-02/02 and three *BaMasc*-05/05 genotypes), and three WZ *BaMasc* hemizygotes (all *BaMasc*-05). (**E**) Violin plots of relative expression between pairs of *BaMasc* zygosity categories (heterozygotes, homozygotes, and hemizygotes), showing contrasting patterns for autosomal (blue; *n*_A_ = 12899) and Z-linked (red; *n*_Z_ = 527) genes. The difference in medians between Z and A is significant for all three *BaMasc* zygosity comparisons (Wilcoxon test with Bonferroni correction: *P* < 0.001).

To test this hypothesis, we first characterized female and male splice variants of *Badsx* from a range of developmental stages. We found one primary variant for each sex (Fig. 3A), plus three additional splice variants weakly expressed in females (fig. S4). As in other Lepidoptera, the male isoform excludes exons 3 and 4 and is shorter than any of the female isoforms (*18*).

We then tested for female isoforms of *Badsx* in *BaMasc* homozygotes in black eggs and larvae collected en masse from the Liverpool stock population at 100 hpo. These experiments, on a sample of 33 black eggs and 29 newly hatched larvae, showed that all *BaMasc* homozygotes (23 black egg embryos with *BaMasc* genotype 03/03, 04/04, or 05/05) express female *Badsx* forms (*Badsx^F^*), and none express the male form (*Badsx^M^*). Conversely, all embryos or larvae heterozygous for *BaMasc* (*n* = 16) expressed *Badsx^M^*, and all *BaMasc* hemizygotes (*n* = 23) expressed *Badsx^F^* (table S6). To avoid potential bias by sampling on phenotype (black eggs), we repeated the assay on younger embryos (69 hpo, before head-capsule darkening) from a line containing only two *BaMasc* haplotypes (02 and 05) and therefore enriched for embryos homozygous at *BaMasc*. As for the older (100 hpo) embryos, all 69 hpo *BaMasc* homozygotes expressed *Badsx^F^* (*n* = 5 each for 02/02 and 05/05), extending the observation that *BaMasc* homozygotes are feminized to a total of four *BaMasc* haplotypes (Fig. 3, B and C, and table S6).

A time series (6 to 100 hpo) of *Badsx* splice forms in the three *BaMasc* zygote categories (fig. S5) revealed that *Badsx^F^* is the dominant isoform in *BaMasc*-hom embryos from 6 hpo, with transient production of *Badsx^M^* at 12 hpo, that becomes barely detectable at 18 hpo. In *BaMasc* heterozygous embryos, expected to develop normally as males, the transition from *Badsx^F^* to *Badsx^M^* is complete by 12 hpo. This transition occurs substantially earlier than in *B. mori* (21 hpo), which develops more slowly, and confirms the view that the default sex at the beginning of development in Lepidoptera is female (*19*). *BaMasc* hemizygous embryos only ever produce *Badsx^F^*.

To examine the effect of zygosity on DC, we analyzed RNA sequencing (RNA-seq) profiles of embryos (69 hpo) from line A (three individuals each of *BaMasc*-02/05, *BaMasc*-02/02, *BaMasc*-05/05, and *BaMasc*-05). The distribution of autosomal gene expression levels was similar between the three genotype categories, with nearly identical medians (Fig. 3D). As in other Lepidoptera (*20*), expression levels of Z-linked genes were adjusted downward in normal ZZ (*BaMasc*-het) males and upward in WZ females, resulting in similar expression levels for Z-linked genes between the sexes despite their different copy number (Fig. 3D). In contrast, the Z-linked gene expression distribution in *BaMasc* homozygotes was visibly right-shifted, relative to *BaMasc* heterozygotes and hemizygotes (Fig. 3D). This indicates a failure of DC in *BaMasc* homozygotes.

The extent of overexpression of Z-linked genes in *BaMasc* homozygotes is made clear by the distribution of relative gene expression for autosomal and Z-linked genes among pairs of *BaMasc* zygosity categories (Fig. 3E). The median *BaMasc*-hom versus *BaMasc*-hem differential expression across the Z chromosome is 1.87, close to, but somewhat less than, the twofold expected if DC in *BaMasc* homozygotes mimicked a female with two Z chromosomes exactly. Median Z:A ratios in *BaMasc*-het and *BaMasc*-hem embryos range from 0.5 to 0.7, depending on the expression level cutoffs applied (fig. S6). Irrespective of the cutoffs, the median Z:A ratio for *BaMasc*-hom embryos is always much higher (~0.9 to 1.3) than for *BaMasc*-het and *BaMasc*-hem (fig. S6). This failure of DC is the most likely cause of embryonic death.

### *BaMasc* initiates sex-specific splicing of *Badsx*

Confirmation that *BaMasc* initiates sex-specific splicing of *Badsx* was achieved by knockdown of *BaMasc* in developing embryos injected with Dicer substrate small interfering RNA (DsiRNA) within the first 4 hpo. The synthetic siRNA molecules targeted two regions: exon 1, including the last two amino acids of the conserved CCCH zing finger (Dsi-01), or exon 5, beyond the masculinizing domain (Dsi-02). The negative control was a DsiRNA designed to not interact with any transcriptome sequences. To avoid sampling dead embryos, individuals were allowed to develop to the black egg stage before being fixed in RNAlater for molecular sexing, *BaMasc* genotyping, and *Badsx* isoform determination (table S7). Survival to the black egg stage was much lower in Dsi-01 (8.96%, *n* = 357) and Dsi-02 (25.6%, *n* = 332) than the control (44.2%, *n* = 224). The sex ratio (WZ: ZZ) at this developmental stage was not significantly affected by the treatments (control *n* = 15:16; Dsi-01 *n* = 17:15; Dsi-02 *n* = 19:13); however, as the embryos were not allowed to hatch and develop, we do not have information on differential viability beyond this point.

**Fig. 4.**
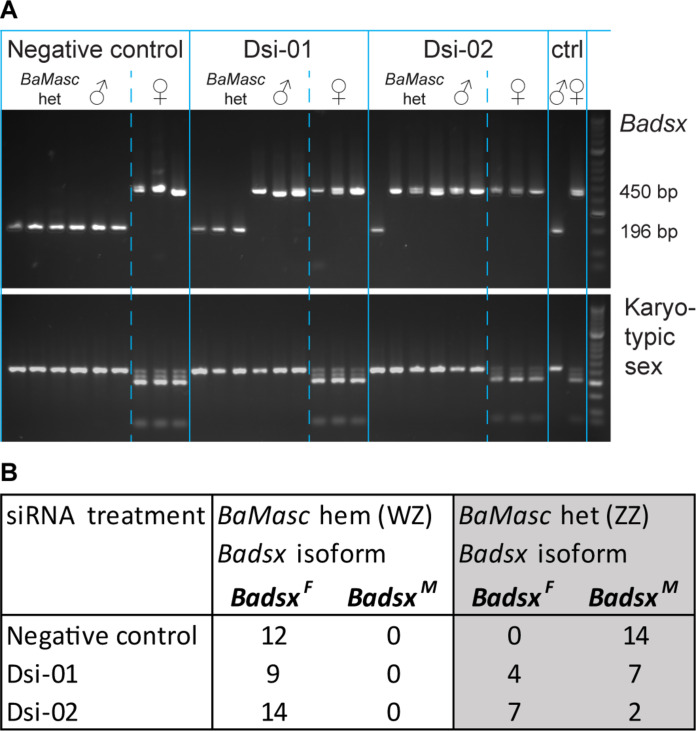
siRNA knockdown of *BaMasc* causes feminization of *Badsx*. (**A**) *Badsx* splice variants (top gel: 196-bp *Badsx^M^* or 450-bp *Badsx^F^*) and karyotypic sex based on W-*BaMasc*/Z-*BaMasc* polymorphisms (bottom gel: ZZ single band or WZ four bands) in a representative sample of six ZZ *BaMasc* heterozygotes and three WZ *BaMasc* hemizygotes from each of three siRNA treatments (negative control, Dsi-01, and Dsi-02). The experimental samples consist of late-stage black egg embryos. The adult (ctrl) samples illustrate the expected patterns for wild ZZ *BaMasc* heterozygous males and WZ *BaMasc* hemizygous females. ZZ *BaMasc* homozygotes and Z0 *BaMasc* hemizygotes were excluded. ZZ *BaMasc* heterozygotes expressing *Badsx^F^* were only observed in Dsi-01 and Dsi-02 and not in the negative control treatment. (**B**) Summary of the experimental results for *Badsx* isoform by siRNA treatment (negative control, Dsi-01, and Dsi-02) and *BaMasc* zygosity (WZ hemizygotes and ZZ heterozygotes), showing that a proportion of the *BaMasc* heterozygotes express *Badsx^F^* in Dsi-01 and Dsi-02 but never in the control sample.

Both of the siRNA treatments produced some ZZ *BaMasc* heterozygotes that expressed the primary *Badsx^F^* isoform rather than the *Badsx^M^* isoform (three in Dsi-01 and seven in Dsi-02), with one additional Dsi-01 individual expressing both of the main isoforms. No such individuals were produced in the control treatment (Fig. 4, A and B). Both of the *BaMasc* knockdown treatments also contain some ZZ *BaMasc* heterozygotes that expressed the standard *Badsx^M^* isoform (more in Dsi-01 than Dsi-02), reflecting variability in the treatments’ effectiveness. The difference between the control and siRNA treatments in the number of ZZ *BaMasc* heterozygotes that expressed the primary *Badsx^F^* isoform (0/14 versus 11/20) is significant (χ^2^: *P* < 0.001). This result demonstrates that disruption of *BaMasc* function in normally viable ZZ males causes them to become transcriptionally female. Very unusually, a small number (4 of 35) of WZ appeared to express both *Badsx^M^* and *Badsx^F^* isoforms, although the *Badsx^M^* band is faint. We attribute this previously unobserved behavior to suboptimal tissue storage and the sensitivity of the end-point PCR.

### *BaMasc* contains a hypervariable region resembling *Apis mellifera csd*

**Fig. 5.**
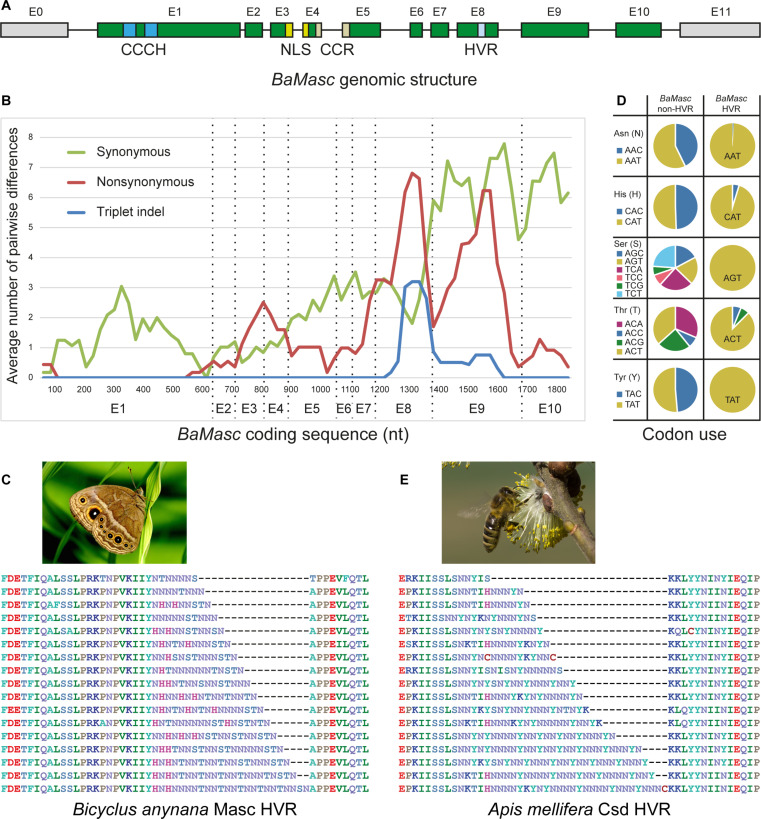
*BaMasc* structure. (**A**) *BaMasc* intron/exon structure (exon:intron size = 20:1), showing the identity of coding exons (green) and 5′/3′UTR (gray) and location of tandem CCCH-type zinc finger domain (CCCH), bipartite nuclear localization signal (NLS), conserved cysteine residues (CCRs) and hypervariable region (HVR). (**B**) Pattern of *BaMasc* coding sequence diversity (99-nt sliding windows with 75-nt overlap) for synonymous and nonsynonymous nucleotide polymorphisms and triplet indels, based on 11 complete haplotypes (proportional differences are shown in fig. S7). (**C**) A sample of partial BaMasc exon 8 amino acid sequences aligned to illustrate length variation in HVR (photo of female *B. anynana* by W. Piel). (**D**) Biased codon usage in *BaMasc* HVR, showing codon usage outside versus inside HVR (*n* = 11 complete haplotypes). (**E**) A sample of amino acid partial sequences of *A. mellifera* Csd exon 7, illustrating length variation in HVR (photo of honeybee by J. Šula, Biology Centre CAS). In (C) and (E), sequences are aligned in the conserved flanks only, with indels in the HVR concatenated and placed at the right-hand boundaries (sequence identifiers are listed in table S8).

To understand which regions within *BaMasc* (Fig. 5A) are important determinants of the homozygous-lethal effect, we explored the pattern of nucleotide diversity across the *BaMasc* coding sequence. We initially examined 11 haplotypes for which the entire gene sequence was available, where we found a sharp increase in sequence diversity in exons 8 and 9, broken down into single nucleotide substitutions (synonymous and nonsynonymous) and indels (Fig. 5B and fig. S7). We then followed up with a targeted survey of exons 8 and 9 in a sample of 246 effectively wild females.

The peak of indel polymorphism occurs within exon 8 (E8) and corresponds to a repetitive hypervariable region (HVR) composed principally of asparagine (N), ranging in size from 7 to 24 amino acids (Fig. 5C). This notable feature has not been previously described in lepidopteran *Masc* sequences (*21*, *22*). Exon 9 of *BaMasc* is less variable in length (94 to 102 amino acids; table S8) but has an exceptionally high level of amino acid polymorphism (69 of 83 variable positions in the sequenced portion, including indels) and nucleotide diversity (π = 0.12 ± 0.01; π_s_ = 0.25 ± 0.03; π_n_ = 0.06 ± 0.01), including occasional (triplet) indels scattered throughout the exon (fig. S8). Despite the high level of indel polymorphism, no frame shifts were detected in E8 to E9. The pattern of nonsynonymous and synonymous variation across the gene is consistent with diversifying selection acting on large parts of the E8 to E9 sequence and purifying selection operating outside of this region.

Close examination of the nucleotide variation and amino acid composition of the HVR implies strong functional constraints. First, its bell-shaped length distribution (fig. S9) resembles that of simple sequence repeats, which are generated through stepwise expansions and contractions, potentially combined with selection at the upper and lower boundaries, and bias toward an optimal length range (*23*). Second, only five amino acids occur in the HVR (N, H, S, W, and Y). Third, the codons for these five amino acids exhibit extreme bias, almost exclusively favoring ANT or NAT codons (Fig. 5D and fig. S10). Codon usage for these five amino acids in the rest of *BaMasc* is not biased (Fig. 5D and figs. S10 and S11). Two of seven amino acids that can be specified by ANT/NAT codons are not used in the HVR (D and I; fig. S11). Thus, while codon usage bias is an expected outcome of replication slippage, the near absence of synonymous (degenerate) point mutations in this long simple sequence repeat suggests an additional purifying process, operating at the level of DNA, RNA, or protein. *BaMasc* HVR bears a conspicuous resemblance to another HVR in *A. mellifera complementary sex determiner* (*csd*), which is also characterized by a variable number of the same five amino acids (plus another three), dominated by N (Fig. 5E) (*4*). The codon usage bias is in the same direction (NAT) for three of the five overlapping amino acids but is generally less extreme (absent for S) and also includes additional codons (NTA/ANA).

The rest of the *BaMasc* sequence structure is similar to that of *B. mori BmMasc* with a tandem CCCH zinc finger motif near the N terminus, followed by a bipartite nuclear localization signal (NLS), and two conserved cysteine residues (CCRs) inside a conserved region in Lepidoptera referred to as the masculinizing domain (*22*, *24*–*26*). The CCCH zinc fingers and the masculinizing domain are completely conserved among the 11 *BaMasc* haplotypes. The NLS is also conserved with only two amino acid substitutions, which do not include any of the four amino acids essential for nuclear localization in *B. mori*. The nonsynonymous polymorphism in the regions containing NLS and CCR (Fig. 5) represents neighboring single-nucleotide polymorphisms (SNPs) within the same sliding window. The tandem CCCH region in *B. anynana* shares 27/51 amino acids with *B. mori*, the NLS only 6/19, and the conserved masculinizing domain 9/11. The domain-defining amino acids (CCCH, KRKK, and CC, respectively) are all identical in *B. anynana* and *B. mori*, showing deep conservation within highly diverged regions for the CCCH and NLS domains (fig. S12). This suggests that the role of the amino acids at these positions is important not only in *B. mori* but also in other Lepidoptera.

### Exceptionally high *BaMasc* haplotype diversity in natural populations

**Fig. 6.**
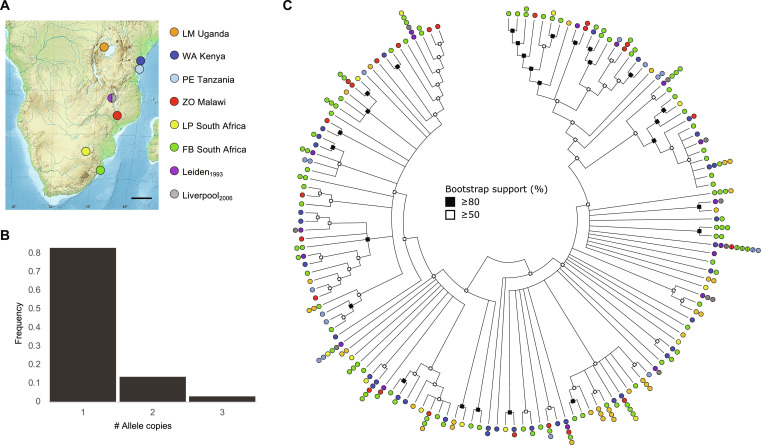
*BaMasc* haplotype diversity in natural populations. (**A**) Geographic origins of samples used in this study. Leiden and Liverpool labels refer to laboratory populations descended from females collected in Malawi in 1988. Scale bar, 500 km. Base map by E. Gaba (Wikimedia Commons user: Sting), CC BY-SA 3.0. (**B**) *BaMasc* (amino acid) allele frequency spectrum in wild female sample, excluding Leiden and Liverpool (*n* = 228). (**C**) Condensed cladogram of the maximum likelihood phylogenetic tree of *BaMasc* E8 to E9 (excluding HVR) 0-fold degenerate sites from an effective sample of 243 females (including any haplotypes shared between Leiden_1993_ and Liverpool_2006_). Nodes with <50% bootstrap support were collapsed. Colored circles indicate the geographic distribution of each partial 0-fold haplotype. For one haplotype, represented 16 times in the sample, half-circles indicate one instance.

Lethality of all *BaMasc* E8 to E9 homozygotes is expected to generate intense negative frequency-dependent balancing selection on *BaMasc* alleles, resulting in a high abundance of rare alleles. This is indeed what we observed in a sample of wild female genotypes collected from southern and eastern Africa (Fig. 6, A and B). More than 80% of *BaMasc* E8 to E9 haplotypes were represented by a single individual, and none were represented more than three times. Across all population samples (including Leiden/Liverpool), we detected a total of 204 different amino acids (205 nt) haplotypes from a total effective sample of 246 females (tables S5 and S8). Extrapolation from the effectively wild female sample (*n* = 228) using the nonparametric iChao1 estimator (*27*) predicts ~750 (95% confidence interval = 567 to 1022) different *BaMasc* amino acid alleles in the global population. However, we emphasize that the number of functionally distinct alleles is unknown, because many haplotypes differing in amino acid sequence are predicted to have the same specificities, forming a smaller set of functional haplogroups (*28*).

Theoretical models predict minimal spatial structuring of loci under balancing selection, relative to neutral loci (*29*, *30*). This is indeed what we find for *BaMasc* E8 to E9, even at a continental scale. The relative similarity of haplotypes between geographically distant populations (including a few shared haplotypes), combined with very high diversity within populations (average haplotype diversity = 0.99), results in low estimates of pairwise differentiation between geographic regions [via analysis of molecular variance (AMOVA): average Φ_ST_ = 0.004 with HVR included, 0.014 with HVR excluded, and 0.015 for synonymous sites with HVR excluded]. This pattern of diversity contrasts with the generally high level of spatial structure of mitochondrial *cytochrome oxidase 1* haplotypes among the same sampling locations (average Φ_ST_ = 0.35) (*31*) in a weakly dispersive species.

Our power to detect phylogenetic relationships among haplotypes was limited by the short length of the sequence data available, as well as homoplasy at both synonymous and nonsynonymous sites. The most informative solution was obtained from 0-fold degenerate sites, excluding the HVR which cannot be reliably aligned (Fig. 6C). This maximum likelihood tree provides modest support for some phylogenetic groupings, mostly at the tips of the tree, but deeper relationships could not be resolved. The highly mixed geographic distribution of many core (0-fold) haplotypes and lineages may be interpreted as reflecting the species-wide spread of *BaMasc* alleles, including repeated reinvasions from neighboring local populations. Selection for newly arisen alleles may also contribute to the overall spatial pattern observed. A haplotype network of the *BaMasc* E8 to E9 amino acid sequences further illustrates the lack of phylogeographic structure at this locus (fig. S13).

The exceptionally high haplotype diversity of *BaMasc* E8 to E9 surpasses that reported for *Apis csd*. The total number of haplotypes recorded for *A. mellifera* and *Apis cerana* are 199 (*32*) and 201 (*33*) amino acid haplotypes, respectively, compared to 204 *BaMasc* haplotypes, including 94 from 104 females in False Bay, SA. The greater diversity of *BaMasc* is also evident from its more extreme allele frequency distribution, characterized by 83% singletons (versus ~43% in *A. mellifera csd*), and a tail truncated at three copies of any given allele within the sample, compared to 11 copies in *Amcsd*. These contrasting patterns imply that the effective population size (*N*_e_) of *B. anynana* has been continuously larger and stable than that of *Apis* spp., whose social system imposes a degree of population substructure, leading to even longer allele retention times, potentially combined with a higher rate of mutation to new alleles. Unfortunately, we did not collect sequence data that would enable us to estimate *B. anynana N*_e_.

Insight into the specific sequence features that determine allele specificities, and therefore compatible pairs (functional heterozygosity), can be gained from controlled crossing experiments of many alleles to identify inviable pairs with non-identical sequence (functional homozygotes) (*34*). However, the limited number of *BaMasc* haplotypes in the contemporary Liverpool stock population, which are all intercompatible, precluded this approach. Moreover, detecting the absence of specific combinations in more diverse populations is impractical because of the very low expected frequencies. Therefore, we explored the crude limits of *BaMasc* functional heterozygosity in the Leiden 1993 sample, which contains 18 *BaMasc* haplotypes, by comparing the observed distribution of genetic distances between the two *BaMasc* alleles (haplotypes) in heterozygous males to a simulated population resampled from the same set of haplotypes. We considered four measures of genetic distance, which focused either on HVR, non-HVR, or the whole E8 to E9 sequence. The shape of the observed distributions of HVR length difference and edit distance are essentially the same as the simulated ones, including the frequency of same length HVR haplotypes (fig. S14, A and B). The non-HVR E8 to E9 Levenshtein and RAxML distance comparisons also support the null hypothesis that all pairs of haplotypes present in Leiden 1993 are compatible (fig. S14, C and D). This does not rule out the possibility that incompatible alleles originally present when the laboratory population was established have been purged. There are no single amino acid positions in the E8 to E9 sequence that are heterozygous in all males (fig. S14E). However, ambiguous alignment may contribute to this result in the HVR region, which is heterozygous as a whole in all males. There are several positions and short regions that are always homozygous.

Closer inspection of the sequence differences among viable haplotype pairs indicates that the minimum HVR edit (Levenshtein) distance within this sample is three amino acid substitutions and/or indels, which occurs in linkage with a minimum of eight amino acid substitutions in the E9 portion of the genotype (fig. S15). Conversely, the most similar E9 sequence, with just two amino acid substitutions, occurs in linkage with eight amino acid differences in the HVR (fig. S15). According to the RAxML distance, the most similar haplotype pair across the whole sequence exhibits a single (six amino acid long) indel and two amino acid substitutions in the HVR, plus one amino acid substitution in E8 (outside HVR) and four amino acid substitutions in E9 (fig. S15). In contrast, the most (RAxML) divergent haplotype pair has 6 amino acid/indel differences in E8 and 15 amino acid/indel differences in E9.

### The W chromosome is not required for female development

The evidence presented thus far for a primary sex determination switch based on *BaMasc* zygosity implies that a W-linked factor, analogous to *B. mori Fem* (*5*), is not required in this system; however, it does not preclude the existence of such a factor or some other role of the *B. anynana* W chromosome in female determination. The serendipitous discovery of the existence of reproductively competent Z0 females, in which the W chromosome is entirely absent, does effectively demonstrate that the *BaMasc* zygosity system functions independently of the W chromosome.

The first karyotype studies of *B. anynana* established that the standard female karyotype is WZ and that the W chromosome is small (*35*). It therefore came as a surprise when a female from an inbred line used in a whole genome sequencing project returned no W-linked sequences (*36*). That line was lost before its significance was appreciated, but a new “Z0 line” was isolated by noninvasively screening females from the same parent stock using W-linked markers.

**Fig. 7.**
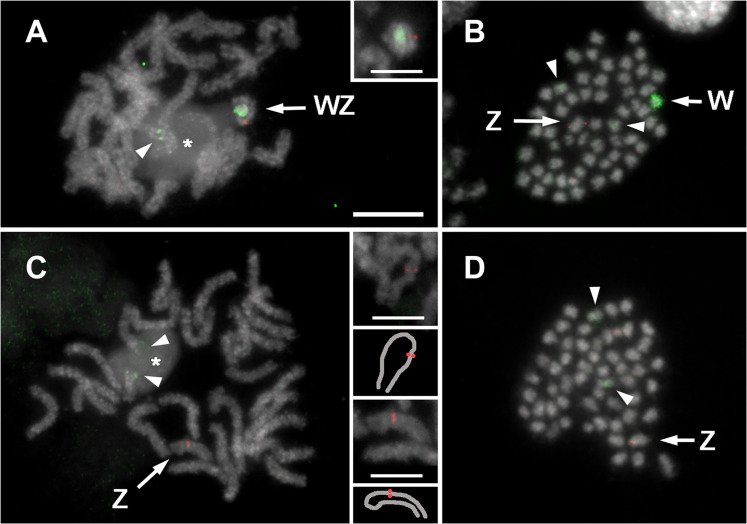
*B. anynana* Z0 females are reproductively competent. Identification of sex chromosomes in WZ (**A** and **B**) and Z0 (**C** and **D**) female larvae by FISH with the W-painting probe (green) and the Z-derived BAC probe (red). Chromosomes were counterstained with 4′,6-diamidino-2-phenylindole (gray). (A) Meiotic chromosomes of a pachytene oocyte with a WZ bivalent. The inset shows a WZ bivalent from another oocyte. (B) Mitotic metaphase with W and Z chromosomes. (C) Meiotic chromosomes of a pachytene oocyte with a U-shaped Z chromosome univalent. The insets show the Z univalent in the main panel (bottom pair) and a Z univalent from another oocyte (top pair). (D) Mitotic metaphase with a single sex chromosome, the Z chromosome. Arrowheads indicate additional hybridization signals of the W-painting probe on an autosomal bivalent associated with the nucleolus [asterisks in (A) and (C)] or on the corresponding pair of autosomes (B and D). Scale bars, 10 μm and 5 μm (insets).

We confirmed the absence of the entire W chromosome in females from the Z0 line by W chromatin staining (fig. S16) and by fluorescence in situ hybridization (FISH) with W- and Z-specific probes (Fig. 7). In WZ line females, the W-painting probe stained the entire W chromosome in meiotic and mitotic nuclei and also stained the autosomal cluster of major ribosomal DNA (rDNA) due to the presence of W-linked rDNA in the probe. The Z-derived bacterial artificial chromosome (BAC) probe identified the Z chromosome as either one strand of the WZ bivalent in pachytene oocytes or as a single element in mitotic metaphases. In contrast, for the Z0 line, no W chromosome was detected with the W-painting probe in meiotic or mitotic nuclei. The Z-BAC probe identified a single, unpaired Z chromosome in these females that often appeared U-shaped, suggesting a tendency to self-pairing. We also applied FISH with the W-painting probe to the remains of a frozen sister of the original putative Z0 female used for genome sequencing (*36*) and confirmed the absence of the W chromosome (fig. S17, A to H). The hemizygous condition of the frozen Z0 female was independently verified by quantitative real-time PCR (qPCR) of two Z-linked genes, which shows that the female had one Z chromosome (fig. S17I). Cytogenetic results were corroborated by negative PCR products for a series of W-linked markers (figs. S18 and S19).

Z0 females, despite lacking a W chromosome, are morphologically indistinguishable from WZ females, pair with males normally, and produce viable offspring. Moreover, they show the female pattern of *Badsx* splicing (fig. S19). Tests for the presence of the W chromosome in population samples using W-markers (*Masc*-W, microsatellites, and rDNA) revealed that Z0 females occur in the wild at ~0.4% (1 of 228) (table S5). We also detected a reproductively competent WZZ male in the Leiden 1993 sample (table S5 and fig. S19), consistent with our conclusion that *BaMasc* zygosity determines sex not the presence/absence of the W chromosome.

## DISCUSSION

We have discovered a primary sex-determining switch for Lepidoptera that functions in a fundamentally different way to the two-locus *Fem*-*Masc* system described for silkworm (*5*). The switch in *B. anynana* consists of different genotypic states: *BaMasc* polyallelism (heterozygotes) versus *BaMasc* monoallelism (hemizygotes and homozygotes) at a single locus. We have shown that, despite most females having a WZ karyotype, the sex-determining mechanism does not rely on a W-linked factor to initiate female development. However, this mechanism cannot distinguish karyotypically male *BaMasc* homozygotes from hemizygotes, leading to an intersex state (male karyotype with female genetic signal), mismatched DC, and death as late-stage embryos.

The observation that degradation of *BaMasc* transcripts through siRNA knockdown in *BaMasc* heterozygotes alters splicing of *Badsx* from the male isoform to the female isoform, while silencing of *BaMasc* in hemizygotes leaves *Badsx* unchanged, supports the idea that heterozygosity (different specificities) of a combination of elements in E8 to E9 confers the active state of the BaMasc protein, whereas hemizygosity (single specificity) or homozygosity (same specificity) represents the inactive state. The inactive state leads to the female pathway by default; the active state presumably forms a heteromeric complex required for male-specific *Badsx* splicing. Our limited analysis found that amino acid differences are required both in the HVR and E9, although we are not able to say with any confidence what the minimum or maximum differences between compatible haplotypes are. The molecular mechanism that distinguishes between the homomeric and heteromeric states of BaMasc remains to be elucidated.

The BaMasc recognition mechanism may resemble the recently described mechanism in the honeybee Csd protein (*37*). In this haplodiploid system, *Amcsd* diploid heterozygotes become viable females, and haploid hemizygotes become viable males, but diploids that are homozygous for *Amcsd* are inviable (eaten by workers). The “active state” of Csd, leading to the female pathway, is a heteromeric complex determined by amino acid differences between alleles within the potential-specifying domain (PSD) that do not bind themselves but direct binding to the conserved coiled-coil domain (CC). The alternative “inactive state” of Csd, leading to the male pathway, is a homomeric complex produced in the absence of amino acid differences within PSD between protein copies (haploid or diploid homozygote), which give rise to PSD binding. Honeybee studies also show that intermediate degrees of specificity between alleles effectively lead to incomplete penetrance, suggesting a potential route through which such complex binary switches may evolve (*34*).

Owing to long retention times, balancing selection at a locus is expected to give rise to functional lineages that are widely distributed at the species level, showing minimal spatial structure (*38*). The lack of spatial structure for *BaMasc* E8 to E9 haplotypes is qualitatively consistent with this expectation, although we did not restrict our analysis to functional lineages. The sequence data available did not allow strong phylogenetic inference, such that less than half of the haplotypes could be reliably grouped, either as terminal branch pairs or larger clades. This pattern contrasts that found in *Apis csd*, also under the same form of balancing selection (negative frequency dependent), in which phylogenetic trees recover well-defined functional lineages (*28*, *39*). The high synonymous nucleotide diversity of *BaMasc* E9 (π_s_ = 0.25 ± 0.03) compared to *Apis* spp. csd-PSD (π_s_ = 0.032 to 0.089), which have *N*_e_ < 10^4^ (*39*), supports the proposition that *B. anynana* has a much larger and stable *N*_e_ and that turnover of *BaMasc* alleles is low. Future work will look to verify this claim using information from field-collected whole genome sequences, which were not available to this study. Interspecific comparisons within *Bicyclus* would also provide valuable insights into the age of the system and allele turnover, although it is likely to be challenging to recover trans-specific alleles or lineages (*40*).

Current natural populations of *B. anynana* rarely experience the recessive lethal cost of this sex-determining system due to the extremely high allelic diversity of *BaMasc*. However, for such a system to evolve in a hypothetical ancestral species with only two *Masc* alleles (equivalent to our line A, with half of all sons dying) must have required strong countervailing selection. A male-killing endosymbiont, such as *Wolbachia*, which infects many *Bicyclus* species (*41*), could plausibly generate such a high cost, as has been demonstrated in other Lepidoptera (*42*–*44*). As male killing can be achieved by targeting *Masc* or other sex determination genes (*45*) and suppressed by their evolution (*46*), the costly features of the *B. anynana* zygosity-based system are consistent with the idea that evolutionary arms races between male-killing bacteria and their hosts are an important driver of diversity in arthropod sex determination systems (*47*). Moreover, under this hypothesis, the *BaMasc* mechanism presents a greater challenge for *Wolbachia* to manipulate than a W-linked feminizing factor because the maternally inherited parasite and the primary switch are not coinherited through the matriline, as daughters receive the Z from their fathers.

The *BaMasc* system adds to the relatively small number of clear cases of single-locus overdominance maintaining genetic diversity through balancing selection (*48*). The form of heterozygote advantage, unconditional lethality of functionally homozygous genotypes, together with large *N*_e_ results in the maintenance of extreme levels of haplotype diversity, characterized by an abundance of rare alleles, through the long-term action of negative frequency-dependent selection (*40*). However, we have been unable to resolve the actual number of functional types underlying the amino acid haplotype frequency distribution. The parallels with *Apis csd* are threefold; first, in the structure of viabilities among hemizygotes, heterozygotes, and homozygotes; second, in the sex switching of homozygotes in the direction of the hemizygous sex; third, in the sequence similarity of the HVRs, which encompasses not only the pattern of trinucleotide repeat length variation but also the strong asparagine bias. While the details of the mechanism in *B. anynana* remain to be determined, these shared features of the HVR would appear to be the result of molecular convergence between two deeply diverged insect lineages to achieve the same outcome: recognition of the same versus different allele specificities (*37*).

## MATERIALS AND METHODS

### Biological samples

All experimental work in this study was performed on captive populations descended from the same Leiden laboratory population established in 1988 from a sample of wild females collected in Malawi by P. M. Brakefield (*11*). No ethics approval was required as the subjects were insects. The exon 8 to 9 haplotype diversity of *BaMasc* was initially characterized using samples from the “Liverpool 2006” and “Leiden 1993” captive populations. We subsequently expanded the sample to include wild adults collected from six localities in southern and eastern Africa in 2007 and 2008 by M. de Jong (*31*). The field collections also gave rise to laboratory-reared generation 1 (G_1_) populations. As the parents of these individuals were not retained for genotyping, we used the mixed offspring populations instead but corrected the *BaMasc* allele frequencies so that each allele within a G_1_ population was only counted once, consistent with the observed distribution in the truly wild samples. This adjustment was necessary to take account of the restricted number of families producing the G_1_ samples, which result in an “effective number” of wild females. The largest female sample (False Bay, South Africa) contains no G_1_ specimens.

In the first experiment to detect *BaMasc* homozygotes (which we had found to be absent in adults), virgin females and males from the Liverpool stock were paired at random to generate a series of full-sib families. A sample of these families, selected based on the *BaMasc* exon 9 haplotype/genotype of the parents, was full-sib mated (individual pairs) to generate broods expected to contain either ¼ *BaMasc* homozygous embryos in the case where parents share a *BaMasc* allele or zero *BaMasc* homozygotes when the parents do not share any *BaMasc* alleles. Eggs were collected over five consecutive days (13.30 to 21.30 each day) from individual females and incubated at 26°C for 5 days, which allows all the embryos that have the capacity to hatch to do so (earliest and latest hatching observed is 84 and 108 hours hpo, respectively). Before freezing, we recorded numbers of hatched larvae, unhatched eggs with visible larval head-capsule (“black” eggs), white uncollapsed eggs (probably fertilized but embryo died before black egg stage), and collapsed eggs (probably unfertilized). Samples of 32 or 48 larvae and unhatched black eggs from 21 full-sib families were genotyped for *BaMasc* exon 9 and molecularly sexed using the W/Z-*Masc* assay. For each of the five focal *BaMasc* haplotypes, the sample for one family was increased to improve the accuracy of segregation estimates.

To facilitate the collection of embryos homozygous for *BaMasc* alleles, we established a line (“line A”) containing only two *BaMasc* haplotypes (02 and 05). All adult males in this line (line A) were *BaMasc*-02/05 heterozygotes, adult females were either *BaMasc*-02 or *BaMasc*-05 hemizygotes, and the sample of zygotes produced by any pairing was expected to contain ¼ *BaMasc* homozygotes (*BaMasc*-02/02 or *BaMasc*-05/05, depending on the maternal haplotype). Staged embryos from this line were used for an RNA-seq experiment and to characterize the expression profiles of *BaMasc* and *Badsx*. In the *Badsx* reverse transcription PCR (RT-PCR) time series (06 to 100 hpo), cDNA from three line A embryos was pooled for each *BaMasc* zygosity category and time point.

The Z0 line, in which females lack the W chromosome, was founded by two Z0 females isolated from the Singapore stock, detected in a random sample of 10 females. The absence of the W in these founding females was determined using a W-linked microsatellite (*49*) on minimally invasive DNA samples extracted from wing clips and subsequently confirmed in descendent generations through the absence of W chromatin body (*35*). Before the creation of this Z0 line, the only material available to verify the existence of *B. anynana* Z0 females was the frozen remains of a sister to three females whose whole genome sequence lacked W-derived sequences. This remnant tissue was used in a first round of cytogenetic and qPCR experiments.

### Genotyping

DNA was extracted using a variety of standard methods, singly or in plate format. Coextraction of DNA and RNA was necessary for some of the embryo/larval samples. Briefly, working on dry ice, egg or first instar larva was placed at the bottom of a 1.5-ml tube with a 3.5-mm steel bead on top and flash spun at 4°C to crush the tissue. Two hundred fifty microliters of TRIzol was added, shaken at 25 Hz using Qiagen TissueLyser II, and left at ambient temperature for 5 min, and 50 μl of chloroform was added and mixed manually by inversion, allowed to stand for 3 min at room temperature, and centrifuged for 15 min at 4°C, 13,000 rpm. One hundred twenty-five microliters of the supernatant was transferred to 1.5-ml LoBind tubes containing 120 μl of isopropanol (remainder of homogenate was kept for DNA extraction) and mixed and centrifuged for 20 min at 4°C, 13,000 rpm. The supernatant was discarded, 500 μl of 75% ethanol was added and centrifuged for 5 min at 13,000 rpm, ethanol was removed and allowed to dry briefly, and RNA was eluted in 35 μl of water. DNA was extracted by transferring all of the remaining homogenate to a 1.5-ml tube containing 180 μl of 100% ethanol and mixed and centrifuged for 15 min at 4°C, 13,000 rpm. The supernatant was removed, and 50 μl of 100% ethanol was added, flash spun, poured off (repeat), air dried, and eluted in 35 μl of Tris-EDTA buffer (TE).

*BaMasc* exons 8 and 9 were genotyped by Sanger sequencing (ABI 3500xL Genetic Analyzer) PCR products. The introns flanking *BaMasc* exons 8 and 9 were too diverse to design reliable primers, which prevented PCR amplification of full-length exonic sequence from genomic DNA. *BaMasc* exons 8 and 9 are highly diverse internally, but the flanks are conserved enough to design degenerate primers based on the 11 available haplotypes. Excluding the primer sequences, the PCRs produce 119 to 170 bp (76 to 82%) of exon 8 haplotype sequence, depending on HVR length, and 219 to 243 bp (78 to 79%) of exon 9 haplotype sequence, depending on triplet indels. PCR and sequencing primers were designed to cost-effectively obtain full-length amplicon sequence from a single Sanger sequencing run (instead of forward and reverse runs). To achieve this, one of the PCR primers of both exon 8 and exon 9 pairs was extended by a tail, which was subsequently used as template for a long Sanger sequencing primer (table S1). As a result, the first fluorescently labeled fragments to pass through the capillary are large enough to produce high Phred quality from the start of the electropherogram. These primer combinations worked for the majority of individuals, but suspected null alleles necessitated the design of multiple primer pairs which targeted different sets of alleles (“PCR failure and allele dropout alternative primer” in table S1). The sequencing objectives were different for wild females and males. For wild females, we focused our effort on obtaining complete haplotype sequence for the amplified regions. In wild males, for whom it was impossible to deconvolute the heterozygous electropherograms, owing to the large number of haplotypes, our aim was to investigate whether *BaMasc* was exclusively heterozygous. In a small number of males with poor quality DNA or suspected allele dropout (7 of 270 individuals), polymorphisms in either exon 8 or 9 (i.e., not necessarily both) were considered evidence for *BaMasc* heterozygosity.

Sequencing electropherograms of *BaMasc* exon 8 and exon 9 heterozygotes in the Liverpool 2006 and Leiden 1993 samples were deconvoluted in Geneious (Biomatters) with reference to a matrix of predicted heterozygous sequence patterns, based on a panel of haplotypes obtained from hemizygous females. The exon 9 sequences for Liverpool 2006 samples were produced with untailed PCR and sequencing primers (Ba_Masc_1387U & Ba_Masc_1649L; table S1) before the tailed PCR sequencing method was routinely used. For family material, to avoid a common indel, a different primer combination was used to amplify a marginally shorter exon 9 fragment (Ba_Masc_1416U to Ba_Masc_1649L), which was then sequenced with a tailed primer (Ba_exon9F_tailed).

In families with a WZ mother, a homozygous *BaMasc* exon 9 genotype was assumed when the Sanger sequencing electropherogram showed no polymorphisms and the molecular sexing indicated the absence of the W chromosome. In contrast, a hemizygous genotype was assumed when the Sanger sequencing electropherogram showed no polymorphisms but the molecular sexing indicated the presence of the W chromosome.

Amplification of *Badsx* from embryo oligo(dT)-primed cDNA using standard RT-PCR was inconsistent. We therefore developed a protocol that accommodates high guanine-cytosine (GC) content, low abundance, templates by combining 7-deaza-2'-deoxyguanosine 5'-triphosphate (7-deaza-dGTP) in the deoxynucleotide triphosphate (dNTP) mix, with random nonamer primed first-strand synthesis, and touchdown thermocycle (*50*). Briefly, cDNA was synthesized using SuperScipt IV reverse transcriptase (Thermo Fisher Scientific) and random nonamers. Each 12 μl of RT-PCR reaction contained the following: 2.4 μl of 5× Green Flexi GoTaq Reaction Buffer (Promega); 0.5 μl of each 10 μM primer (Ba_dsx_479U and Ba_dsx_656L; table S2); 1.2 μl of 10× deaza-dNTP mix [2 mM deoxyadenosine triphosphate, 2 mM deoxycytidine triphosphate, 2 mM deoxythymidine triphosphate (dTTP), 0.5 mM dGTP, 1.5 mM 7-deaza-dGTP (Sigma-Aldrich, D8783)]; 0.72 μl of 25 mM MgCl_2_; 0.45 μl of GoTaq Hot Start polymerase (Promega); 6 μl of water; and 1 μl of cDNA template. The thermocycle, performed on a Veriti PCR machine, was 94°C for 2 min, followed by 40 cycles of 94°C for 30 s, 68°C for 30 s (reducing by 0.3°C each cycle to 56.3°C), and 72°C for 40 s; followed by 15 cycles of 94°C for 30 s, 60°C for 30 s, 72°C for 40 s; and a final extension of 72°C for 2 min.

### Recombination mapping to define the homozygous lethal interval

A PCR-based Z-linkage map with 29 distantly spaced markers (table S1) was created using the 2BF_1_ family following previously described procedures (*11*). The homozygous lethal interval was coarsely mapped to 1.4 Mb. The father and six sons from the 2BF_1_ family were whole genome sequenced to a median mapped coverage of 10× using a NEBNext Ultra II FS library prep kit for Illumina (with 3 PCR cycles to minimize duplicates). Four offspring were used to phase polymorphisms and confirm the PCR-based recombination matrix. Two individuals were used to fine-map the recombination breakpoints which define the lethal interval. Mapping polymorphisms were obtained by read mapping against the reference sequence using Geneious version R10.

### *BaMasc* copy number and lethal interval genic heterozygosity determination

The haploid single-copy presence of *BaMasc* was determined by blastn and tblastn searches in the primary and alternate haplotype ilBicAnyn1.1 assemblies, which represent the same single female. *BaMasc* has a single representative in the primary ilBicAnyn1.1 assembly and is absent in the alternate as would be expected for a single-copy Z-gene in a hemizygous genome assembly pair. In addition, the coverage of *BaMasc* short reads in male BaM_17127D and female BaF_17127D DNA sequencing (DNA-seq) reads represented two (diploid) and one (hemizygous) copy, respectively. *BaMasc* polymorphisms are inherited as single-copy Z alleles (paternal to son and daughter, maternal exclusively to sons). Exons of *BaMasc* with distinct polymorphisms were found in female BaM_17127D DNA-seq reads. These were confirmed to be W-linked by exclusive mother to daughter inheritance and absence in males. The absence of transcriptional activity in any RNA-seq and smallRNA sequence runs available in the Sequence Read Archive database suggests that these are pseudogenized copies. The estimated *W-BaMasc* exon copy number varies between 1 and 13 based on female BaM_17127D DNA-seq read coverage compared with their Z counterparts (table S2).

The genomes of 31 adult males were short-read sequenced to a median mapped coverage of 8× using a NEBNext Ultra II FS library prep kit for Illumina (New England Biolabs), with 3 PCR cycles to minimize duplicates. One additional male (BaM_17127D) was sequenced to higher mapped coverage (65×) using the Nextera XT PCR-free protocol. All the males were sampled from different subpopulations of the Liverpool laboratory population (10 from 2017 and 22 from 2023). The reads from individual BaM_17127D was trimmed using fastp (0.23.4) (*51*). Other reads were provided as trimmed pairs by the sequencing provider. The reference scaffold corresponding to the Z chromosome was manually curated within the coarsely mapped lethal region (NC_069110.1 5,599,186 to 7,453,263) to fill gaps in the scaffold using bridging PacBio reads belonging to PRJEB54937. The read sets were then mapped to the modified reference genome, using bwa-mem (0.7.17-r1188). Mapped reads were subsequently filtered for MAPQ > 30 and sorted with samtools (1.18) and then filtered for duplicates with picard tools (2.8.1). Variants were called from the mapped bam files with freebayes (1.3.6) with alternate alleles limited to 4 (--use-best-n-alleles 4). To filter the called genotypes to nonsynonymous sites, we produced a bed file of sites from the results of the predictCoding function implemented in the R (4.2.2) package VariantAnnotation (1.44.1). We extracted sites from the vcf according to this bed file using bedtools intersect (2.31.0). Last, we identified the absence of homozygous genotypes at the exon and gene level, using an R script wrapped around vcfR (1.14.0), GenomicFeatures (1.50.4), and ggplot2 (3.4.2) libraries.

### Molecular sexing assay

The molecular sexing assay is based on PCR–restriction fragment length polymorphism (PCR-RFLP) using the variation between W- and Z-representatives of *BaMasc* exon 1. PCR amplification of W- and Z-*Masc* exon 1 with a primer pair matching both was followed by a W-specific *Taq*I restriction digest and visualization on 2% agarose gel. This method produces unambiguous results for all variants found in lab strains, and all but one of the wild populations were studied. The W-sequence has two *Taq*I sites, one of which also occurs frequently in Z-*Masc* exon 1 of the Lake Mburo population. Molecular sexing of the Lake Mburo population requires sufficiently long electrophoresis runs with controls present to allow correct interpretation of the bands. In addition, this particular Lake Mburo haplotype produces a 6-bp shorter PCR fragment due to a deletion of two prolines. This 6-bp size difference is too small to be distinguished on a 2% agarose gel. Often, undigested or partially digested fragments remain after 3 hours of *Taq*I incubation, producing “unpredicted” bands. Increasing the incubation time usually does not result in full digests either. Increasing the enzyme concentration (also increasing the per-reaction cost) was not considered necessary because the presence of partial digests does not affect the distinctiveness between females and males (fig. S1).

PCR conditions are as follows: 12 μl of reactions containing 400 nM each primer Ba_Masc_131U and Ba_Masc_532L (table S1), 300 μM each dNTP (BioLine), 1× LongAmp buffer, 1.2 U of LongAmp *Taq* DNA polymerase (New England Biolabs), and 1 μl of 100× H_2_O diluted genomic DNA. Cycling conditions are as follows: 95°C denaturation for 3 min followed by 40 cycles of 30 s at 95°C, 30 s at 60°C, and 45 s at 70°C. Restriction digestion are as follows: 16 μl of reactions containing 1× CutSmart buffer, 3 U of *Taq*I (New England Biolabs), and 7 μl of the PCR product. Incubation was for 3 hours at 65°C followed by separation on a 2% agarose gel.

### Reference sequences for *BaMasc* and *Badsx*

The coding sequence of *BaMasc* was obtained by a tblastn search using the translated *B. mori Masc* sequence (GenBank AB840788) against an unreleased draft *B. anynana* assembly. Only exon 1 shared enough similarity between the two species to return a blast hit. Putative exons 2 and 3 were found in the scaffold containing *BaMasc* exon 1 with Augustus gene prediction (University of Greifswald) implementing the *Heliconius melpomene* model. Predicted exons 1 to 3 were confirmed by pooled-egg cDNA PCR and Sanger sequencing. The exon 1 to 3 region was extended beyond the stop codon by 3′ rapid amplification of cDNA ends based on pooled-egg RNA to produce a draft complete cds made up of mixed haplotypes. The draft complete cds was used to obtain haplotypes from DNA sources (short-read DNA-seq raw data, BACs, and genomic DNA PCR–Sanger sequencing) and RNA sources (RNA/cDNA PCR–Sanger sequencing, and short-read RNA-seq raw data). BACs 40D22 and 53H3, containing haplotypes BaMasc_011 and BaMasc_017, respectively, were identified by the Clemson University Genomics Institute using overgo probes designed from *BaMasc* exon 1 sequence. The BAC DNA was isolated with the BACMAX DNA Purification Kit (Epicentre Biotechnologies).

Initially, only female sequences were used to take advantage of unambiguous phasing in Z hemizygotes. Subsequently, heterozygous male haplotypes in short sequence reads could be deconvoluted (i.e., phased) once one of the haplotypes was obtained from females. Distinct polymorphisms throughout the cds were used to identify the different haplotypes. Female short RNA-seq reads belonging to the same sequencing project were pooled per haplotype to obtain sufficient read depth. This was necessary because a proline-rich region in exon 10 is often severely underrepresented and error-prone due to extreme GC content. Reads were mapped to the draft complete cds using Geneious version R10 with short k-mer lengths (16 nt) and large error tolerance (20%) taking the high diversity of *BaMasc* into account. Consensus sequences were manually corrected in positions where nonspecific reads mapped as a result of the relaxed error tolerance. Reads were remapped iteratively with lower error tolerance to the consensus sequences to verify the correct assembly of the haplotypes. Haplotypes H_liv_01, H_liv_05, BaMasc_006, BaMasc_008, BaMasc_011, and BaMasc_017 were obtained from National Center for Biotechnology Information (NCBI) PRJNA376691 RNA-seq reads. The exon 10 GC-rich region was poorly covered in BaMasc_011 and BaMasc_017 and was therefore confirmed by Sanger sequencing this region in BACs 40D22 and 53H3. Haplotypes H_liv_02, H_liv_03, and H_liv_04 were obtained by PCR and Sanger sequencing of cDNA from 100 hpo embryos Livs_0417_100hpo_31, Livs_0417_100hpo_32, and Livs_0417_100hpo_38, respectively. BaMasc_010 was found in three male runs of NCBI bioproject PRJEB10924 and deconvoluted by subtracting H_liv_02 and BaMasc_017. Low coverage regions were improved by PCR and Sanger sequencing of st93fem_076, which shares the BaMasc_010 haplotype. BaMasc_015 was only found in NCBI biosamples SAMEA3245886 and SAMEA3546073, which are both males. The BaMasc_015 haplotype was deconvoluted by subtracting BaMasc_006 and BaMasc_008, respectively. The SAMEA3245886 DNA reads were mapped to separate *BaMasc* exons instead of complete coding sequence to avoid intron-exon boundary mismatches. Low-coverage regions were confirmed by PCR and Sanger sequencing of st06 female 37, which carries the same haplotype. This whole exercise resolved 11 distinct complete haplotypes (NCBI OR085802 to OR085812). Eight of these were confirmed independently in separate genome assemblies or RNA-seq data.

*Badsx* coding sequences were first manually predicted on the basis of genomic sequence and then confirmed using mRNA sequence. One male-specific and four female-specific splice variants were found. A tblastn search with *B. mori Bmdsx^F^* (AB048543) and *Bmdsx^M^* (AB048544) found parts of *Badsx* exons 1 and 5 and complete exons 2, 3, and 4 in an unreleased draft *B. anynana* genome assembly. The exon 5 prediction had to be improved using similarity with *Helicoverpa armigera dsx^M^* (KF419131) because the last 31 amino acids of the *B. mori* AB048544 sequence are incorrect. BACs containing *Badsx* were identified by the Clemson University Genomics Institute using an overgo probe targeting *Badsx* exon 2. A gap in exon 1 was closed by Sanger sequencing BAC 54i18 (table S1). The predicted *Badsx^F^* sequence was confirmed using RNA-seq data from bioproject PRJNA376691 which is made up exclusively of females. These sequences were also used to identify UTRs. The predicted male splice variant was confirmed with PCR and Sanger sequencing (table S1) using cDNA from 99 hpo male embryos. Female PCR products often contained secondary bands that were larger than the common 450-bp *Badsx^F^* fragment. Samples with strong secondary bands were Sanger sequenced and deconvoluted by extracting the sequence of the common 450-bp fragment. The deconvoluted sequences were aligned to the genome assembly to verify the sequence and to establish the intron-exon boundary positions. This revealed that larger variants of exons 3 and 4 exist with alternative splice donor and acceptor sites, respectively, as is the case in *B. mori dsx* exons 3 + a and b + 4 (*18*). All four possible combinations of common and larger variants of exons 3 and 4 were found in 99 hpo female cDNA sequences, obtained by Sanger sequencing RT-PCR products, and were subsequently inferred from PCR fragment sizes on gels. The five splice variants are available in NCBI (OR085813 to OR085817).

### *BaMasc* sequence diversity analyses

The 11 complete *BaMasc* haplotypes were aligned using “translation align” in Geneious version R10, producing a 1875-nt alignment covering 624 amino acids plus the stop codon. The alignment (DOI: 10.5281/zenodo.10419704) is gapped due to single or multiple triplet indels in exons 8 and 9. To explore patterns of polymorphism within the coding sequence, diversities were calculated for sliding windows of 99 nt (75-nt overlap), separately for synonymous and nonsynonymous SNPs, and for indels. The Nei-Gojobori method (*52*), implemented in MEGA version 11 (*53*) with the pairwise gap deletion option, was used to calculate the average number and proportion of pairwise synonymous and nonsynonymous nucleotide differences. Indels, which always occur in multiples of three nucleotides, were treated as triplet indels. Correspondingly, the proportion of indels was defined as the average number of pairwise indels per triplet or amino acid length (i.e., 33 triplets in a 99-nt window). The implicit assumption that the 11 complete haplotypes occur at equal frequencies is consistent with the observed haplotype frequency distribution for a much larger sample of *BaMasc* exons 8 to 9 haplotypes. Nucleotide diversities were also estimated from 246 effectively wild partial sequences of *BaMasc* exon 9 (80% complete), which can be aligned reliably (DOI: 10.5281/zenodo.10419704). Maximum composite likelihood method was used for total nucleotide diversity (π) and the Nei-Gojobori (p-distance) for synonymous (π_s_) and nonsynonymous (π_n_) nucleotide diversities, with all SEs calculated by the bootstrap procedure (100 replicates).

Codon usage in *BaMasc* HVR was compared to codon usage in *BaMasc* coding sequence excluding HVR (*BaMasc* non-HVR), based on the 11 complete *BaMasc* haplotypes. A Python script was used to extract triplet codons for the HVR and non-HVR portion of each haplotype. Counts for each of the 61 possible triplets were obtained in Excel and converted to haplotype-specific proportions of total codons in HVR or non-HVR. The same approach, restricted to HVR, was used for the 204 haplotype set.

The conserved domains in *BaMasc* were identified by amino acid sequence similarity with *B. mori BmMasc* for the CCCH zinc-fingers and the CCRs and the wider masculinizing domain. The bipartite NLS was identified with PSORT II prediction (https://psort.hgc.jp/) and extended by one upstream amino acid to include all three lysines (K) and the arginine (R) essential for nuclear localization in *B. mori*.

An estimate of the total number of *BaMasc* alleles that exists across the entire species distribution was obtained using an approach that estimates the asymptote of a “species” (allele) accumulation curve from the lower-order frequency counts (*54*). We chose Chao1-type estimators which are nonparametric in the sense that they make no assumptions about the mathematical form of the underlying distribution. As our data contained singletons, doubletons, and tripletons, we applied the iChao1 estimator, which is more accurate than the original Chao1 estimator that only uses singletons and doubletons (*27*). The reported estimate and confidence intervals were calculated with SpadeR (*55*, *56*) on the pooled (effectively) wild sample of females (*n* = 228). Pooling samples from demographically isolated populations is justified given the very low levels of differentiation. Technically, the estimate obtained represents the lower bound but may be considered as a satisfactory point estimate when the frequencies of rare alleles are nearly homogeneous, which is the case here (*54*).

### Phylogenetic and population structure analysis

Phylogenetic analysis of *BaMasc* haplotypes began by concatenating exons 8 and 9, separated by a short stretch of 6 N nucleotides. The full sequence was aligned using the codon-aware MACSE (v2.03) alignment program (*57*). Extracting the maximum phylogenetic signal from the sequences required dealing with a HVR within exon 8. This region cannot be aligned with high confidence due to a multitude of indels and indel length polymorphisms and low complexity recurring amino acid residues. To account for this, we followed methodology implemented by Lücking *et al.* (*58*). First, the HVR-aligned region was excised, and a distance matrix was calculated with Ngila (*59*), which aims to improve the scoring of gap costs and indel lengths. We then applied the PICS-Ord R script, which converts this distance matrix into scaled integer values via principal coordinates analysis. The integer-coded HVR and well-aligned amino acids could then be combined into a single distance matrix with RAxML (v8.2.9) (*60*) using the Mk model for the HVR multistate partition and the PROTGAMMA model for the amino acid partition (comprising the non-HVR portion of exon 8 and the entirety of exon 9). We imported the resulting distance matrix and aligned sequence (DOI: 10.5281/zenodo.10419704) with the R package poppr (v2.8.6) and used it to calculate a minimum spanning network (*56*, *61*). Because of the extreme haplotype diversity, minimum spanning network graphs with scaled branch lengths were not possible to plot in an interpretable layout. We therefore opted to set branch lengths to a fixed distance and used the “layout_with_lgl” function implemented in the R package igraph, which is tailored to resolving large graphs.

A more stringent approach to reconstructing phylogenetic relationships among the haplotypes excluded the HVR and extracted 0-fold degenerate sites from the MACSE alignment with MEGA (version 11.0.11). Sequences were collapsed to singletons using seqkit (version 2.7.0). The extracted sites were then used as the input for the model finding function of IQ-TREE (version 2.2.6), outputting the TIM2e+G4 model. This was subsequently run with 10,000 ultrafast bootstraps, and the resulting tree was collapsed to a cladogram, collapsing nodes with <50% bootstrap support with MEGA. Individuals with shared alleles were annotated onto the tree manually.

We also used poppr for calculating analysis of molecular variance statistics (using the pegas implementation and 10,000 permutations) for all polymorphisms in the concatenated exons 8 and 9 sequence (using the RAxML distance matrix), for all polymorphisms excluding HVR (defined by the presence of >6-bp indel variations), and for fourfold degenerate synonymous polymorphisms only, excluding the HVR.

### Functional heterozygosity

Patterns of within-individual sequence difference between *BaMasc* haplotypes in population samples of adult (i.e., developmentally viable) heterozygous males can, in principle, provide insights about the determinants of functional heterozygosity in this system. As the wild male samples could not be genotyped to the level of constituent haplotypes (because of too high diversity), the analysis of pairwise differences was conducted on the Leiden 1993 sample only (table S3). Even in this reduced *BaMasc* diversity population (18 haplotypes, giving 144 potential heterozygous combinations), a sample of 110 males is inadequate to properly test the hypothesis that certain heterozygous combinations are inviable, but it is useful for examining the distribution of pairwise genetic distances between haplotypes in viable heterozygotes (two genotyped individuals, st93male_59 and st93male_77, were excluded from this analysis). We used four measures of genetic distance between *BaMasc* exons 8 to 9 amino acid haplotypes: (i) absolute length difference of the HVR; (ii) Levenshtein distance of the unaligned HVR; (iii) Levenshtein distance of the aligned exons 8 to 9 sequences, excluding HVR; and (iv) the inclusive RAxML distance described above. To contextualize the observed pairwise distances, we produced a simulated set of 1000 males for comparison using a Python script (DOI: 10.5281/zenodo.8060179). This was achieved by repeatedly sampling pairs of haplotypes at random from the haplotype frequency distribution contained in the observed male sample. If the haplotypes were the same (homozygous), then the simulated individual was discarded.

### RNA-seq experiment

To test whether aberrant gene expression played a role in the homozygous *BaMasc* lethal phenotype, we performed an RNA-seq experiment in late-stage embryos collected from line A, which only contains two *BaMasc* haplotypes (02 and 05). We selected the 69 hpo (26°C) time point as a compromise between ensuring sex determination had been initiated and avoiding widespread cell death due to the lethal phenotype observed in *BaMasc* homozygotes. *BaMasc* genotype and karyotypic sex were determined using Sanger sequencing and PCR-RFLP assay, respectively, using coextracted DNA/RNA samples. cDNA was synthesized from the associated coextracted RNA samples. cDNA from individual embryos was polyA-selected [oligo (dT)_25_ magnetic beads] before library preparation with the NEBNext Ultra II Directional RNA Library Prep Kit for Illumina (fragmentation for 15 min at 94°C). The overall experimental design consisted of three biological replicates per condition. Four conditions were assessed: hemizygous females (05), heterozygous males (02/05), homozygous males (02/02), and alternate homozygous males (05/05). The 12 cDNA libraries were sequenced as paired reads (2 × 150 bp) on one lane of a HiSeq 4000 (Illumina Inc.).

Reads were trimmed by the sequencing provider, as follows. The raw Fastq files were trimmed for the presence of Illumina adapter sequences using Cutadapt (version 1.2.1) with option -O 3, so that the 3′ end of any reads which match the adapter sequence for 3 bp or more is trimmed. The reads were further trimmed using Sickle (version 1.200) with a minimum window quality score of 20. Reads shorter than 15 bp after trimming were removed. To quantify gene expression, we used a published genome assembly for *B. anynana* (GCF_947172395.1) and its associated gene annotation.

Pseudo-count data were generated using Salmon (version 1.9.0) (*62*). The gene annotation used was judged to be conservative as the RNA mapping rate to this transcriptome was fairly low (34 to 45%), whereas a de novo annotation with StringTie (*63*) provided a much higher mapping rate. The overall observations reported are consistent between both analyses; however, the precise values of Z:A ratio vary according to the annotation used.

We imported the Salmon output into R using tximport (version 1.22.0), collapsing transcript counts to the gene level, and calculated abundance with the option “lengthScaledTPM.” To demonstrate the effect of low expression and nonexpressed genes in the comparisons, we used a cutoff approach, similar to previous investigations in other species (*64*). Selection of specific filtering criteria can alter Z:A expression ratio estimates; therefore, for transparency, we present all combinations of minimum sample number (1 to 12) and minimum expression values [>0, >1, and >2 transcripts per million (TPM)] in fig. S6. On the basis of this analysis, the criteria for inclusion were an expression value > 1 TPM in at least three samples.

Expression comparisons between pairs of *BaMasc* zygosity categories, including quality checks (dispersion and volcano plots), were performed using the DESeq2 (version 1.34.0) package (*65*, *66*). For presentation purposes, we also truncated the *y* axis, excluding a small number of outliers. A more conservative interpretation of expression differences applied a log_2_ fold change shrinkage function, which reduces outlier values when the overall amount of information for a given gene is low. This gave a median Z-linked differential expression between *BaMasc*-hom and *BaMasc*-hem of 1.6. Wilcoxon nonparametric test of median differences was performed using the compare_means function and the plotting functions of ggpubr R package.

### *BaMasc* knockdown using siRNA

The experiment used the Singapore stock of *B. anynana*. Larvae and adults were reared in a temperature-controlled room at 27°C and 65% relative humidity with a 12:12-hour light:dark photoperiod. The caterpillars were fed on maize leaves and adults on banana.

The *BaMasc* haplotype sequences were aligned using MUSCLE in MEGA (gap open, −400; gap extend, 0; max iterations, 16). Two DsiRNAs were designed using the Integrated DNA Technologies’ (IDT) RNAi design tool to target highly conserved regions in the *BaMasc* haplotypes. DsiRNA 1 (Dsi-01) targets the 155- to 180-bp region of *BaMasc* (exon 1, including the last two amino acids of the conserved CCCH zing finger), and DsiRNA 2 (Dsi-02) targets the 970- to 995-bp region (exon 5, beyond the CCR/masculinizing domain). These sequences (Table 1) were checked for specificity using the BLAST tool in LepBase (*67*, *68*).

**Table 1.** Nucleotide sequences of DsiRNA and universal negative control.

Dsi-01 (155–180 bp)	Sense strand	AGUGCAAGUAUCGUCAUGAACUUGT
	Antisense	ACAAGUUGAUGACGAUACUUGCAGUGU
Dsi-02 (970–995 bp)	Sense strand	GAUGAGUACGAACAGCUAAAAGAAA
	Antisense	UUUCUUUUAGCUGUUCGUACUCAUCAA
Negative control	Sense strand	CGUUAAUCGCGUAUAAUACGCGUAT
	Antisense	AUACGCGUAUUAUACGCGAUUAACGAC

Batches of embryos to be injected were collected on maize leaves from the stock population within 3 hpo and transferred to petri dishes. Embryos were injected within 45 min of collection (i.e., within 4 hpo). The DsiRNA or universal negative control (IDT, negative control DsiRNA) was resuspended with nuclease-free water (to a final concentration of 100 μM) and diluted with an equal volume of nuclease-free duplex buffer (50 μM working solution). Nontoxic blue food dye was added to the injection mixture for easy visualization (0.2 μl was added to 5 μl of DsiRNA mixture). We used borosilicate glass capillaries (World Precision Instruments, 1B100F-3) pulled with a micropipette puller (Sutter Instrument Co., model P-97). Embryos were injected using an Eppendorf FemtoJet 4i according to the protocol in (*69*).

After injection, a moist cotton pad was placed in each petri dish to prevent the embryos from dessicating. The petri dishes were kept at 25°C, and the injected embryos were checked twice daily for development. Once the embryos reached the “blackhead” stage (between 72 and 120 hpo), in which the black head capsule of the developing larvae can be seen, the chorion was broken with fine forceps to extract the developing embryo and place it in RNAlater (Thermo Fisher Scientific). Embryos in RNAlater were left overnight at 4°C and then transferred to −20°C. This tissue storage procedure, rather than freezing the whole egg at −80°C, was necessary for logistical reasons, as the subsequent processing of the samples was conducted in a different laboratory.

Individual embryos were rinsed in phosphate-buffered saline to remove RNAlater before RNA-DNA coextraction, as described in the “Genotyping” section. DNA was used for molecular sexing and *BaMasc* exon 9 genotyping, and RNA was used for *Badsx* end-point RT-PCR (see the “Genotyping” section). As the Singapore stock was known to contain a low frequency of Z0 females, a small number of embryos that could not initially be sexed unambiguously based on the presence/absence of W, because they lacked W and either expressed female *Badsx* isoform or it was undetermined (PCR failure), were measured for Z dosage using genomic qPCR, with *Tan* as the Z-linked gene and *Aos1* (*activator of SUMO 1*) as the autosomal gene (two Z0 were detected in the whole sample, with one additional ZZ/Z0 embryo remaining undefined). We attribute the higher rate of *Badsx* amplification failure in the whole sample of 101 embryos (26%), compared to samples frozen at −80°C, to partial RNA degradation affecting the integrity of this weakly expressed gene in some embryos.

### Cytogenetics

Meiotic chromosomes were obtained from testes and ovaries of fourth and fifth instar larvae, respectively. Mitotic chromosomes were obtained from either gonads or wing imaginal discs of the same specimens of both sexes. Slides with spread chromosomes were prepared as previously described (*70*). Briefly, tissues were dissected in physiological solution, swollen in hypotonic solution (75 mM KCl) for 8 to 10 min, fixed in Carnoy’s fixative (ethanol, chloroform, acetic acid, 6:3:1) for 10 to 30 min, dissociated in 60% acetic acid, and spread on the slide using a heating plate at 45°C. Ovaries were mostly fixed without hypotonic treatment to preserve the chromomere pattern of pachytene bivalents. Preparations were then passed through an ethanol series (70, 80, and 100%; 30 s each) and stored at −20°C until further use. Shortly before use, selected preparations were removed from the freezer, dehydrated in the graded ethanol series, and air-dried.

Polyploid interphase nuclei for sex chromatin assay were obtained from Malpighian tubules of fifth instar larvae of both sexes. Tubules were dissected out in physiological solution, briefly fixed in Carnoy’s fixative, and stained in 1.5% lactic acetic orcein. The preparations were examined under a light microscope for the presence of female-specific sex chromatin, which is formed by multiple copies of the W chromosome (*35*, *71*).

To obtain preparations of interphase nuclei from frozen pupae of the standard (Liverpool) laboratory population and from the frozen remains of the putative Z0 pupa, specimens were removed from the freezer and slowly thawed in cold physiological solution. As their organs were degraded, small pieces of unspecified tissues were fixed directly on the slide in Carnoy’s fixative for about 3 min, dissociated in 60% acetic acid, spread using a heating plate at 45°C, and further processed as described for chromosome preparations.

To prepare a W chromosome painting probe for *B. anynana*, we obtained W chromosome DNA by laser microdissection of sex chromatin bodies (i.e., W chromatin) from highly polyploid cells of Malpighian tubules following the previously published protocol (*72*) with slight modifications (*73*). Briefly, Malpighian tubules were dissected from the last instar female larvae in physiological solution, swollen in hypotonic solution (75 mM KCl) for 10 min, fixed in methanol-acetic acid (3:1) for 15 min, transferred into a drop of 60% acetic acid on a thin glass slide coated with polyethylene naphthalate membrane (Goodfellow, Huntingdon, UK), spread using a heating plate at 40°C, and stained with 4% Giemsa (Penta, Prague, Czech Republic). Microdissection of sex chromatin bodies was performed using a PALM MicroLaser System (Carl Zeiss MicroImaging, Munich, Germany) as described previously (*74*). PCR amplification of W chromosome DNA and probe labeling were performed as previously described (*73*). About 15 sex chromatin bodies per sample were amplified using the GenomePlex Single Cell Whole Genome Amplification Kit (WGA4, Sigma-Aldrich), and then the reaction was purified using the Wizard SV Gel and PCR Clean-Up System (Promega). The amplified product was labeled using the GenomePlex WGA Reamplification Kit (WGA3, Sigma-Aldrich). The labeling reaction mix contained 10 ng of amplified DNA, 0.4 mM each dNTP except 0.33 mM dTTP, 40 μM Cy3-dUTP or 40 μM ATTO 488-dUTP (both Jena Bioscience), 1× Amplification Master Mix, and 1.25 μl of WGA polymerase in a total volume of 18.75 μl.

We selected a BAC clone 4F1 from the *B. anynana* BAC library constructed by the Clemson University Genomics Institute as a Z chromosome marker. The BAC clone 4F1 originates from the Z chromosome and contains a Z-linked *C5197* gene (GenBank accession no. KC996762) (*75*), which is located in close proximity to the *BaMasc* gene. To prepare a Z-BAC probe, BAC-DNA was extracted using the BACMAX DNA Purification Kit (Epicentre Biotechnologies). The BAC-DNA was fragmented by heating at 99°C for 4 min and amplified using the WGA4 kit. Subsequently, the BAC-DNA fragments were reamplified with the WGA3 kit and labeled by nick translation with a mixture of deoxyribonuclease I and DNA polymerase I (both Thermo Fisher Scientific) with Cy3-dUTP (Jena Bioscience).

FISH using W-painting and Z-BAC probes was performed according to previously published protocols (*76*, *77*) with some modifications. Slides were denatured at 68°C for 3.5 min in 70% formamide in 2× SSC buffer. Probes were denatured at 90°C for 5 min. To test the quality of the W-painting probe and for FISH with the W-painting probe on interphase nuclei of frozen pupae, the probe cocktail for one slide contained the W-painting probe precipitated from 5 μl of the labeling mixture and 25 μg of sonicated salmon sperm DNA in a total volume of 10 μl of 50% formamide and 10% dextran sulfate in 2× SSC. For the two-color FISH to demonstrate the existence of Z0 females, the probe cocktail was similar but contained an additional 450 ng of the Z-BAC probe and 5 μg of fragmented unlabeled male genomic DNA as a competitor. Slides were hybridized with the probe cocktail at 37°C for 3 days. Slides were then washed at 62°C (FISH with W-painting probe) or 42°C (two-color FISH) for 5 min in 0.1× SSC containing 1% Triton X-100 and counterstained with 4′,6-diamidino-2-phenylindole (0.5 μg/ml; Sigma-Aldrich) in antifade based on DABCO [1,4-diazabicyclo(2.2.2)-octane; Sigma-Aldrich]. FISH preparations were observed in a Zeiss Axioplan 2 microscope (Carl Zeiss) equipped with a monochrome charge-coupled device camera XM10 (Olympus), and images were captured separately for each fluorescent dye using cellSens Standard software version 1.9 (Olympus). Images were pseudocolored, merged, and optimized for brightness and contrast using Adobe Photoshop CS6.

To test whether the frozen putative Z0 female pupa had a single Z chromosome, we compared the relative doses of two Z-linked genes between samples of frozen pupae by qPCR using genomic DNAs as templates according to a previously described method (*78*). Genomic DNA was extracted from the frozen female pupa (WZ), the frozen male pupa (ZZ), and the frozen remains of the putative Z0 pupa using the standard phenol-chloroform procedure. We selected the Z-linked *B. anynana* orthologs of *NBAD hydrolase* (*Ba_tan*; GenBank accession no. KC996755) and *epidermal growth factor receptor* (*Ba_torpedo*; GenBank accession no. KC996754) as target genes and an ortholog of the *activator of SUMO 1* (*BaAos1*; GenBank accession no. XM_024084177) gene as an autosomal reference gene. Primer sets for the target and reference genes are listed in table S1. The qPCR experiment was performed exactly according to the procedure described elsewhere (*79*). Briefly, the reaction mixture contained 0.5 to 20 ng of template genomic DNA, 0.5 pmol of each primer, and 5 μl of Xceed qPCR SG 2× Mix Lo-Rox (Institute of Applied Biotechnologies, Prague, Czech Republic). For calculation of amplification efficiency (E), four points of a fourfold dilution series were used. The data obtained were processed using CFX Manager Software (Bio-Rad). The PCR reaction was performed using the C1000 Thermal Cycler CFX96 Real-Time System (Bio-Rad). The female-to-male relative dose ratio of the target gene was determined by comparison with the autosomal reference gene using a published formula (*80*) and statistically analyzed by Welch’s *t* test with a Bonferroni correction for multiple testing.

### W chromosome markers

A total of 11 W-markers in four genomic regions were discovered in various ways as described below, followed by a standardized validation which tested whether the markers were inherited exclusively from mothers to daughters as proof of W-linkage. A cross of two randomly selected virgin parents from the Liverpool stock population and eight offspring of each sex were used for this purpose. All W-markers described here produced complete sex-specific segregation (present in mother and daughters, absent in father and sons), which corresponds with a Fisher exact probability test *P* value of 0.000155. All W-markers were amplified using the same conditions as described for the molecular sexing assay. PCR products were either run on agarose gels or Sanger sequenced for genotyping. The PCR and sequencing primers can be found in table S1.

The gene *courtless* mapped to the end of the Z chromosome using paternal SNPs in a mapping family (*11*). Maternal SNPs in the same region of *courtless* were exclusively inherited by daughters. Unfortunately, these SNPs were also present as allelic variants on some Z chromosomes and were therefore unsuitable as universal W-markers. However, the mapping cross did demonstrate that a paralog of *courtless* exists on the W chromosome. Genome assemblies available at the time of investigation were poorly resolved in the Z-chromosomal *courtless* region, and a W-assembly is still lacking. A search for a better *courtless* W-marker in genomic short sequence reads found that part of exon 4 occurs in several copies in the intron between exon 4 and exon 5, both on the Z chromosome as well as in the putative W-sequences. These partial exons contain a stop codon and are not included in mRNA and are therefore referred to as “pseudoexons” (cf. pseudogenes). The reverse primer was designed in one of the pseudoexon 4 regions believed to be W-specific, and the expected result was that females would produce a 150-bp band while males would produce none. The target 150-bp band indeed only shows up in females (fig. S18); there are however numerous additional bands both in females and in males, including a second female-specific band with an estimated size of 370 bp. The recent Darwin Tree of Life Z-chromosome assembly (ilBicAnyn1.1) allowed us to reinterpret the results. It reveals that *courtless* is extremely close to the telomere (23 kb from 3′UTR), that seven (tandemly repeated) copies of pseudoexon 4 exist, and that the reverse primer sequence which was initially thought to be W-specific is also found once on the Z chromosome. This information explains the banding pattern seen on the gel for males and also provides a likely explanation why previous assembly efforts failed in this region. The W chromosome is not assembled for *B. anynana*, but the female-specific banding pattern clearly shows that a similar repetitive structure consisting of pseudoexon 4 copies must exist on the W chromosome. The difference in banding pattern between males and females reveals that the arrangement of the repetitive components must be different. Despite this genomic complexity, the *courtless* PCR produces unambiguous distinctive patterns between males and females and serves as a reliable W-marker.

One microsatellite from a panel of 28 was found to be W-linked (*81*) (GenBank accession AY785080). The original published primers were replaced with a better performing pair (table S3). The microsatellite W-marker uses the presence/absence of PCR bands to distinguish between females and males (fig. S18). This can potentially lead to false negatives as a result of unsuccessful female PCR wrongly interpreted as males. However, the results were consistent with those of the other W-markers when using the redesigned W-microsatellite primers.

A cytogenetic study showed that a nucleolar organizer region (NOR), where ribosomal RNA is mass-transcribed from rDNA, is associated with the W-Z bivalent in *B. anynana* (*35*). We explored whether at least part of the NOR originated from the W chromosome by examining differences between genomic male and female short-read sequences. Three polymorphisms were confirmed to be W-linked by exclusive mother to daughter inheritance, thus providing evidence that rDNA is present on the W chromosome. These polymorphisms require Sanger sequencing because they are unsuitable for gel-based PCR-RFLP or indel assays.

Sequences homologous to *BaMasc* exons 0 to 5 were found exclusively in female genome short reads. Their presence on the W chromosome was confirmed by exclusive mother to daughter inheritance of W-specific polymorphisms. Each exon gave rise to one marker (totaling six). An indel differentiating the W and Z variants of the noncoding 5′UTR *BaMasc* exon 0 was used to produce PCR amplicons to be separated on gels (fig. S18). *BaMasc* exon 1 W and Z representatives are described in detail in the “Molecular sexing assay” section and fig. S3. The remaining four *W-BaMasc* exons (2, 3, 4, and 5) were genotyped by Sanger sequencing.

### Verification of putative Z0 females using W chromosome markers

W chromosome loss by means of aneuploidy resulting in Z0 instead of WZ females was hypothesized when a W-linked microsatellite sequence (*81*) was not found in female genomic sequence reads (NCBI bioproject PRJEB8426). Further support for this hypothesis was generated by confirmation of the sex (because wrongly sexed males also lack W chromosomes) and the absence of additional W-linked markers. Conclusive evidence of W-loss was later provided cytogenetically.

Unlike adults, which are easily sexed by distinct external morphological features, sexing of pre-adult stages is more involved and may require confirmation by different methods. Pupae can be reliably sexed by the presence of testes or ovaries after dissection, but the pupae used for bioproject PRJEB8426 were sexed by external morphological characteristics instead, which are sometimes misinterpreted. Molecular sexing based on the W-marker presence or absence (i.e., the molecular sexing assay) cannot be used for Z0 females. Therefore, the confirmation of sex relied on the zygosity of *BaMasc* exons 8 and 9, which is hemizygous in females and always heterozygous in postembryonic males as a result of embryonic homozygote lethality. In addition, genomic read depth of Z-linked genes versus autosomal genes, which have an expected Z:A ratio of 1:1 in ZZ males and 2:1 in WZ or Z0 females, was used to confirm the sex of the bioproject PRJEB8426 females. For this purpose, we mapped reads to exons larger than 1 kb belonging to single-copy genes which had been confirmed by linkage mapping to be either on Z- or autosomal chromosomes (*11*, *75*). This produced Z:A coverage ratios between 1:2.05 and 1:2.11, thereby confirming that the morphologically categorized bioproject PRJEB8426 females were hemizygous for Z. Investigating the presence or absence of additional W-markers was based on the same W-linked polymorphisms described in the W chromosome markers section, using read mapping instead of molecular techniques. None of the W-polymorphisms were found in the bioproject PRJEB8426 females, thereby strengthening support for the hypothesis that these females did not have a W chromosome. RNA extracted from the pupal remains of a sister of the bioproject PRJEB8426 females was tested for *Badsx* splice variant and found to conform to the standard female pattern.

One female from the wild collection (an280 from Lake Mburo) and two females from the Leiden 1993 captive population (st93fem_03 and st93fem_10) tested negative for the W-*BaMasc* exon 1 polymorphism and were investigated further to establish whether these are Z0 females. In the absence of any diagnostic body parts, verification of sex was based on *BaMasc* exons 8 and 9 zygosity and Z:autosomal ratio genomic qPCR, using *Tan* as the Z-linked gene and *Aos1* (*Activator of SUMO 1*) as the autosomal gene (wild an280 only). The presence or absence of the W chromosome was tested using the 11 polymorphisms as described in the “W chromosome markers” section. The three females matched all the Z0 criteria (fig. S19A), with the exception of the untested *Badsx* splice form, as no RNA was available, and the untested Z:A ratio for the two Leiden 1993 females. In addition, one male from the Leiden 1993 captive population was positive for the W-*BaMasc* exon 1 polymorphism. Testing the same markers as the three Z0 females showed the presence of all W-markers and heterozygosity of *BaMasc* exons 8 and 9. This combination suggests that this male has WZZ sex chromosomes, adding further evidence that the W chromosome is not involved in sex determination.
