# Supplementary material for: Zygosity-based sex determination in a butterfly drives hypervariability of Masculinizer
Source: Sci Adv. 2024 May 3;10(18):eadj6979. doi: 10.1126/sciadv.adj6979 (PMC11067997; doi:10.1126/sciadv.adj6979)
Supplement: Supplementary file 1 — Figs. S1 to S19 Legends for tables S1 to S8 Legend for movie S1 [file sciadv.adj6979_sm.pdf]

Science Advances
AAAS

Supplementary Materials for

Zygosity-based sex determination in a butterfly drives hypervariability of *Masculinizer*

Arjen E. van't Hof *et al.*

Corresponding author: Ilik J. Saccheri, saccheri@liverpool.ac.uk

Sci. Adv. **10**, eadj6979 (2024)
DOI: 10.1126/sciadv.adj6979

The PDF file includes:

Figs. S1 to S19
Legends for tables S1 to S8
Legend for movie S1

Other Supplementary Material for this manuscript includes the following:

Tables S1 to S8
Movie S1

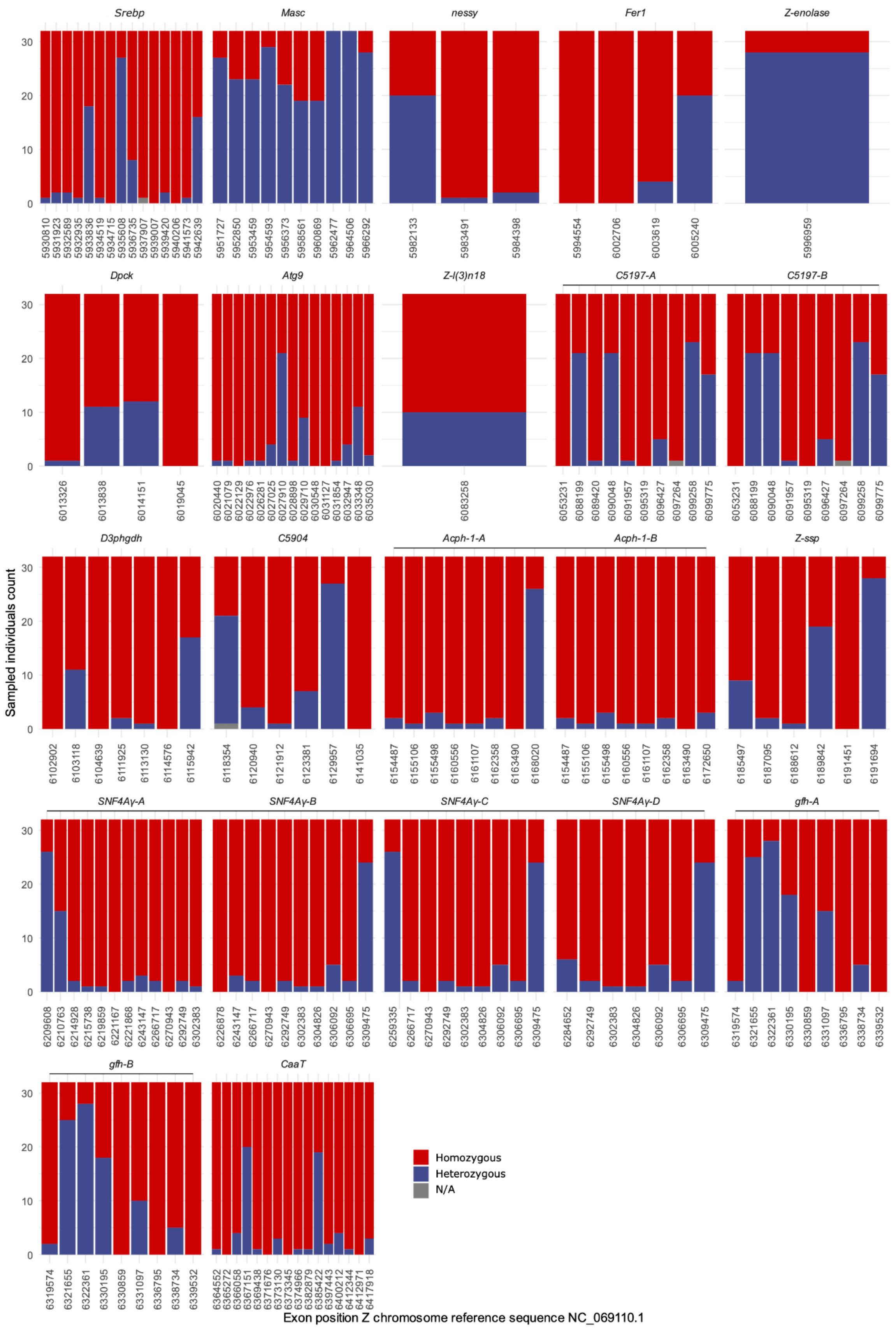
Srebp
5930810 5931923 5932589 5932935 5933836 5934519 5934715 5935608 5936735 5937907 5939007 5939420 5940206 5941573 5942639
Masc
5951727 5952850 5953459 5954593 5956373 5958561 5960869 5962477 5964506 5966292
nessy
5982133 5983491 5984398
Fer1
5994554 6002706 6003619 6005240
Z-enolase
5996959
Dpck
6013326 6013838 6014151 6019045
Atg9
6020440 6021079 6022129 6022976 6026281 6027025 6027910 6028898 6029710 6030548 6031127 6031854 6032947 6033348 6035030
Z-l(3)n18
6083258
C5197-A
6053231 6088199 6089420 6090048 6091957 6095319 6096427 6097264 6099258 6099775
C5197-B
6053231 6088199 6090048 6091957 6095319 6096427 6097264 6099258 6099775
D3phgdh
6102902 6103118 6104639 6111925 6113130 6114576 6115942
C5904
6118354 6120940 6121912 6123381 6129957 6141035
Acph-1-A
6154487 6155106 6155498 6160556 6161107 6162358 6163490 6168020
Acph-1-B
6154487 6155106 6155498 6160556 6161107 6162358 6163490 6172650
Z-ssp
6185497 6187095 6188612 6189842 6191451 6191694
SNF4Aγ-A
6209608 6210763 6214928 6215738 6219859 6221167 6221868 6243147 6266717 6270943 6292749 6302383
SNF4Aγ-B
6226878 6243147 6266717 6270943 6292749 6302383 6304826 6306092 6306695 6309475
SNF4Aγ-C
6259335 6266717 6270943 6292749 6302383 6304826 6306092 6306695 6309475
SNF4Aγ-D
6284652 6292749 6302383 6304826 6306092 6306695 6309475
gfh-A
6319574 6321655 6322361 6330195 6330859 6331097 6336795 6338734 6339532
gfh-B
6319574 6321655 6322361 6330195 6330859 6331097 6336795 6338734 6339532
CaaT
6364552 6365272 6366058 6367151 6369438 6371676 6373130 6373345 6374966 6382879 6385422 6397443 6400212 6412344 6412971 6417918
0 10 20 30
Sampled individuals count
Exon position Z chromosome reference sequence NC_069110.1
Homozygous
Heterozygous
N/A

Fig. S1. Exonic heterozygosity in *BaMasc*-containing lethal interval

The number of adult *Bicyclus anynana* males out of a sample of 32 (sampled from partially inbred laboratory populations) that were amino acid homozygous (or heterozygous) for each exon in the sixteen genes (and their isoforms, grouped by horizontal lines) present within the Z chromosome interval that was found to be homozygous lethal in backcross families.

		F_1 ZZ father *BaMasc* genotype	
		$Z_1\ Z_2$	$Z_1\ Z_3$
F_1 WZ mother *BaMasc* genotype	W Z_2	½ $Z_2\ Z_1$<br>½ $Z_2\ Z_2$	½ $Z_2\ Z_1$<br>½ $Z_2\ Z_3$
	W Z_3	½ $Z_3\ Z_1$<br>½ $Z_3\ Z_2$	½ $Z_3\ Z_1$<br>½ $Z_3\ Z_3$

Fig. S2. Expected proportions of ZZ offspring genotypes

Punnett square showing the expected proportions of *BaMasc* genotypes in the ZZ (normally male) progeny of the four possible crosses between the full-sibs produced by a parental cross in which the mother's *BaMasc* allele is different to either of the father's ($WZ_1 \times Z_2Z_3$). If the F_1 mother's *BaMasc* allele is the same as either of the F_1 father's alleles then half of the sons are expected to be homozygous (grey shading); otherwise all sons are expected to be heterozygous (green shading).

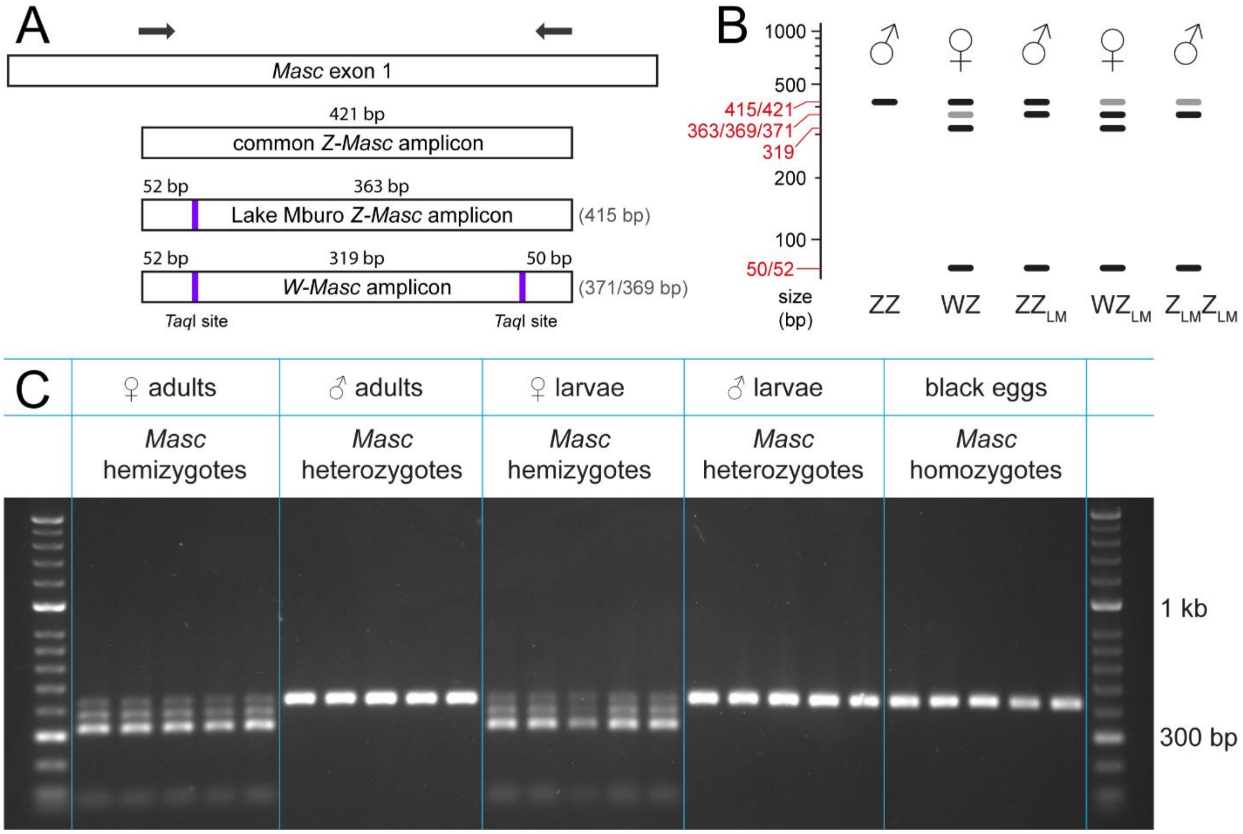

Fig. S3. *Bicyclus anynana* molecular sexing assay

(**A**) The position of the PCR amplified region relative to the W and Z representatives of *BaMasc* exon 1, and the *Taq*I restriction sites in the different variants (purple). PCR primer positions are indicated by the arrows, the digest fragment sizes are shown above each amplicon (partial digest sizes to the right). (**B**) Predicted product sizes for both sexes including the Lake Mburo haplotype (Z_{LM}). The figure is redrawn from REBSites (https://tools.neb.com/REBsites/) using the amplicon sequences in combination with *Taq*I digest and 2% agarose gel. Black bands represent the full digests, grey bands partial digests. Similar-sized overlapping bands are shown as single bands as they would appear on a gel. (**C**) Restriction digest of females and males for different developmental stages and *BaMasc* zygosity. Female adults and larvae show the undigested Z band (421 bp), the W digest (319 bp and overlapping 52 and 50 bp), and the overlapping partially digested 371 and 369 bp bands. The karyotypic males only produce undigested 421 bp bands. *BaMasc* homozygosity (rather than hemizygosity) of Sanger sequenced unhatched black eggs was established by the presence of the undigested Z band and absence of W digest. The digest pattern is independent of the specific *BaMasc* exon 8-9 haplotype, as illustrated by the inclusion of five different haplotypes (01–05), within each sex/stage category, in the example gel.

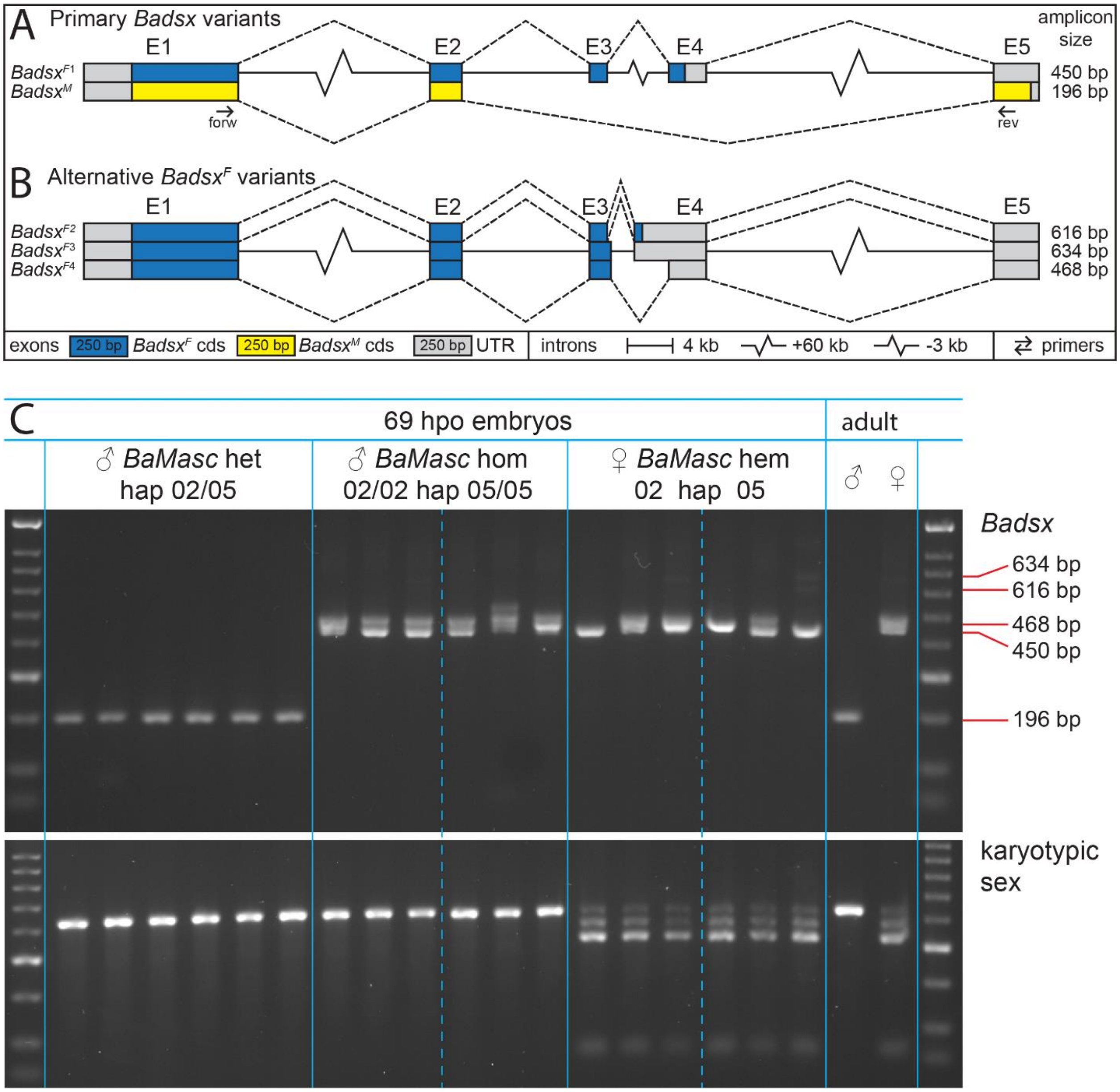

Fig. S4. *Bicyclus anynana doublesex* splice variants

(**A** and **B**) Intron-exon structure of the various *Badsx* splice variants, with solid lines representing introns, blue and yellow rectangles exonic coding sequence of female and male variants, respectively, grey boxes untranslated regions (UTRs), and dotted lines connecting the exons included in the different variants. The key includes scale boxes for exons and scale bars for introns. (A) The primary variants of *Badsx* for each sex (also shown in Fig. 3). (B) Additional *Badsx*^{F} splice variants found exclusively in females and *BaMasc* homozygous karyotypic males. These alternative splice variants use alternative splice donors and acceptors for exons 3 and 4 respectively, resulting in different sized transcripts. (**C**) The upper gel image shows *Badsx* splicing bands produced under different types of *BaMasc* E8–9 zygosity (heterozygous (het), homozygous (hom), hemizygous (hem)) in 69 hpo embryos, with adult male and female splicing included for reference. Heterozygous males produce the *Badsx*^{M} isoform, while homozygous

karyotypic males and hemizygous females produce the different *Badsx*^{F} isoforms. The *Badsx*^{F4} variant occurs frequently and is moderately expressed, as shown by the presence and intensity of the 468 bp band. The two larger bands (616 and 634 bp), representing *Badsx*^{F2} and *Badsx*^{F3}, are seen faintly but clearly in the 6th female and very faintly in a few other samples. The 5th homozygous male contains a band which probably represents a heteroduplex, not showing the fragment's actual size. The lower gel image shows the digest pattern of molecular sexing, confirming that homozygous males only produce variants of *Badsx*^{F}, while heterozygous males and hemizygous females produce the expected sex-specific splice variants.

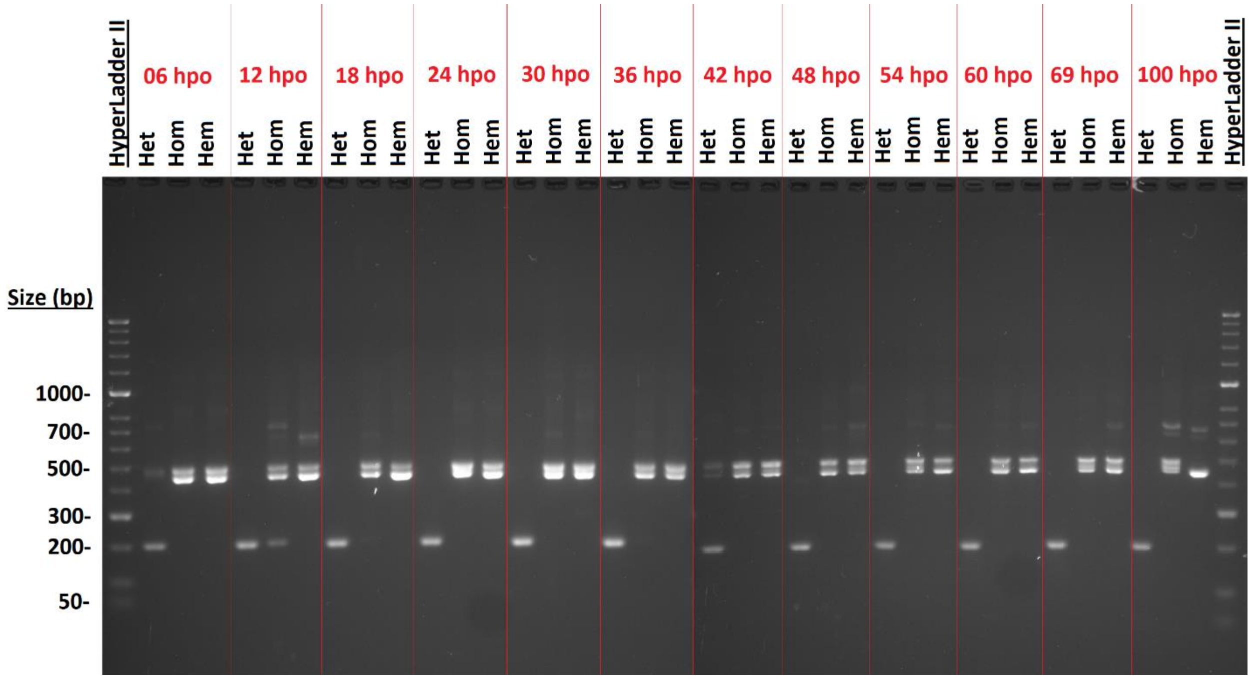

Fig. S5. *Badsx* isoform expression in developing embryos

Badsx end-point RT-PCR time series (06-100 hpo), showing the expression of female (*Badsx*^{F}) and male (*Badsx*^{M}) splice variants in three *BaMasc* exon 8-9 categories of developing embryos of *Bicyclus anynana*: heterozygous ZZ males; homozygous ZZ males; and hemizygous WZ females. *Badsx*^{M} is indicated by the single band at 196 bp; *Badsx*^{F} are the larger fragments starting at 450 bp. The faint *Badsx*^{F} bands in 42 hpo het may be due to contamination.

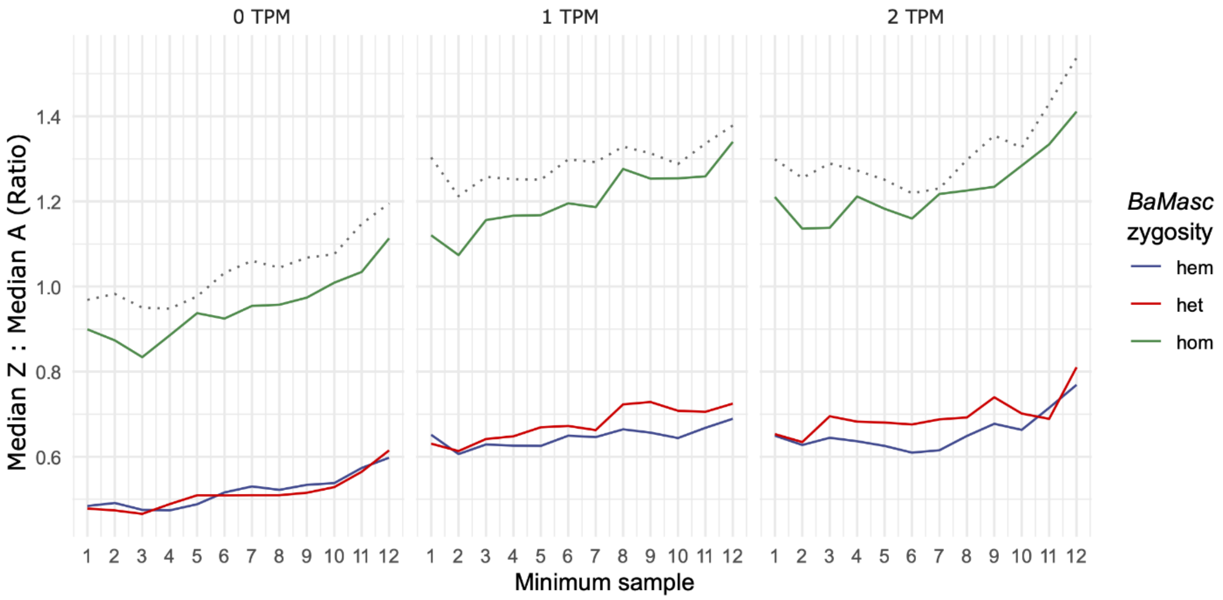

Fig. S6. *Bicyclus anynana* Z:A ratio

The combined effect of minimum expression and minimum sample threshold criteria on the value of Z to autosome (Z:A) expression ratio in *Bicyclus anynana* 69 hpo embryos. The x-axis corresponds to the minimum number of samples for which a minimum TPM (transcripts per million) expression is observed. Three values for minimum TPM (0, 1 and 2) are shown in the different plot facets. Overall, the filtering criteria is least stringent on the leftmost data point (must have > 0 TPM in at least one sample) and most stringent on the rightmost datapoint (must have > 2 TPM in all 12 samples). Zygosity defines samples that are either hemizygous (hem), heterozygous (het) or homozygous (hom) at *BaMasc* exon 8–9. For reference, we include a dotted line showing double the value observed in hemizygous females, which represents a hypothetical value of female-typical dosage up-regulation applied to a male-typical ZZ karyotype.

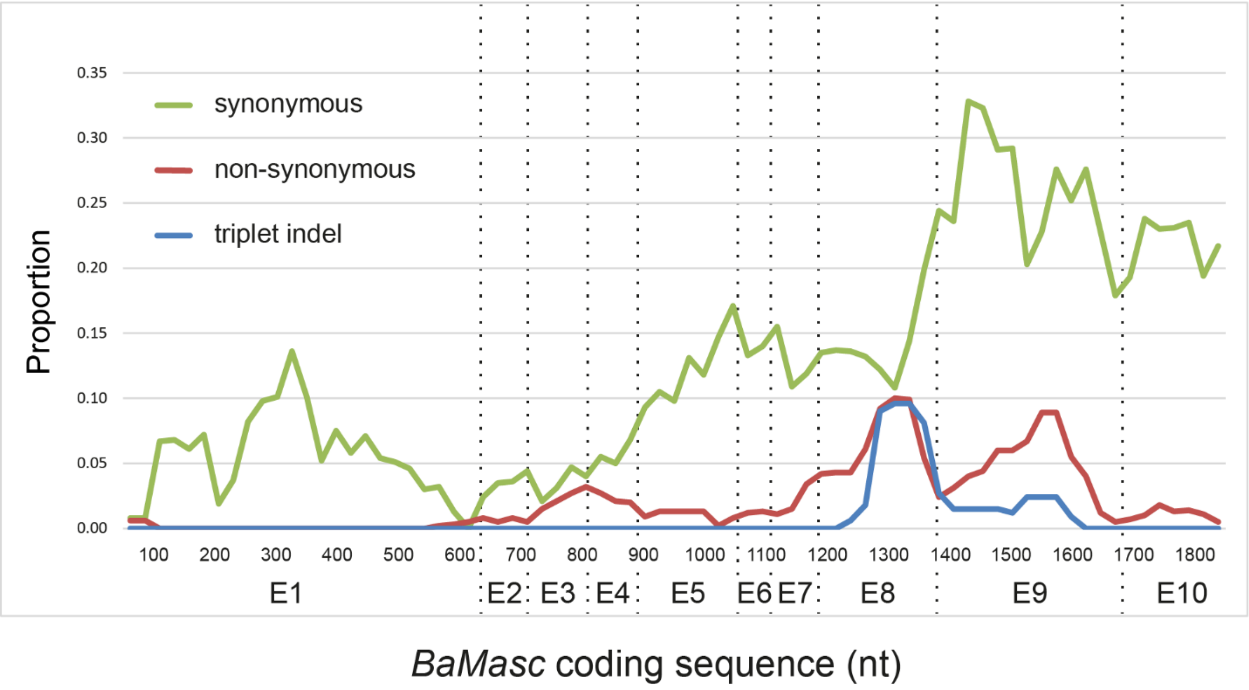

Fig. S7. *BaMasc polymorphism*

Sliding window pattern of coding sequence diversity across all ten exons (E1–E10) of *BaMasc*, based on a sample of 11 full haplotypes. Proportions of the average number of pairwise differences are shown for synonymous (π_s) and non-synonymous (π_n) SNPs, and triplet indels. Windows are 99 nt wide with 75 nt overlap. π_s and π_n were calculated with respect to the corresponding numbers of sites; the triplet indel proportion with respect to the total number of triplet sites in the window (33 triplets). Values should be treated as conservative, particularly for non-synonymous SNPs and triplet indels, as they are based on a small number of sequences. Due to the different mode of mutation (replication slippage), the SNP proportions for E8-HVR cannot be interpreted as π_s and π_n.

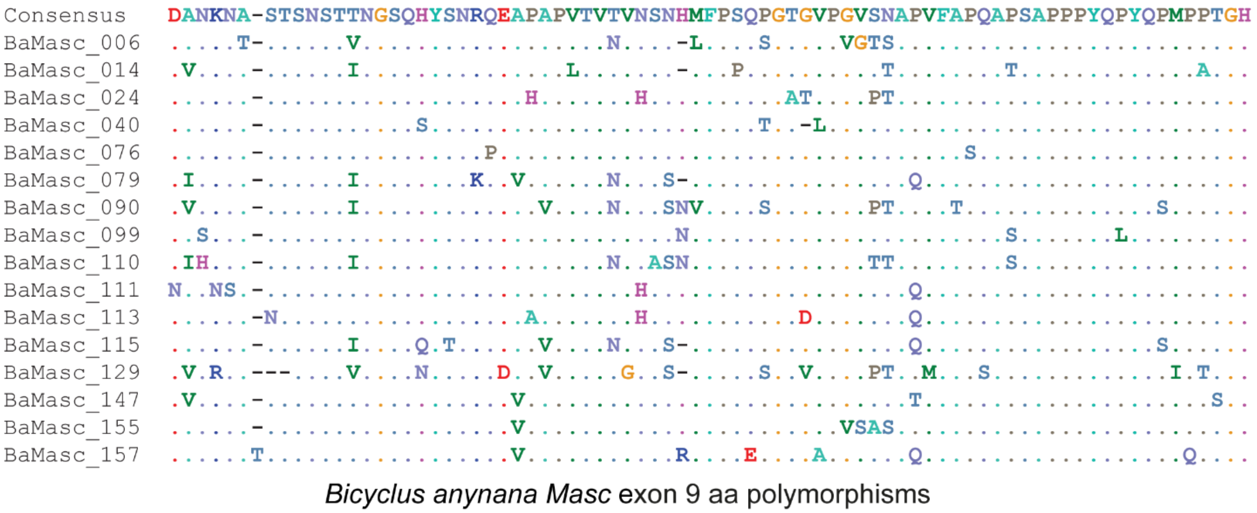

Fig. S8. Amino acid polymorphism among haplotypes of *BaMasc* exon 9

Illustrative *BaMasc* exon 9 aa alignment for a sample of sequences showing extensive substitution and indel polymorphism. The sequences show translated primer-trimmed amplicons which exclude 12 and 9 aa, respectively, from the beginning and end of the exon.

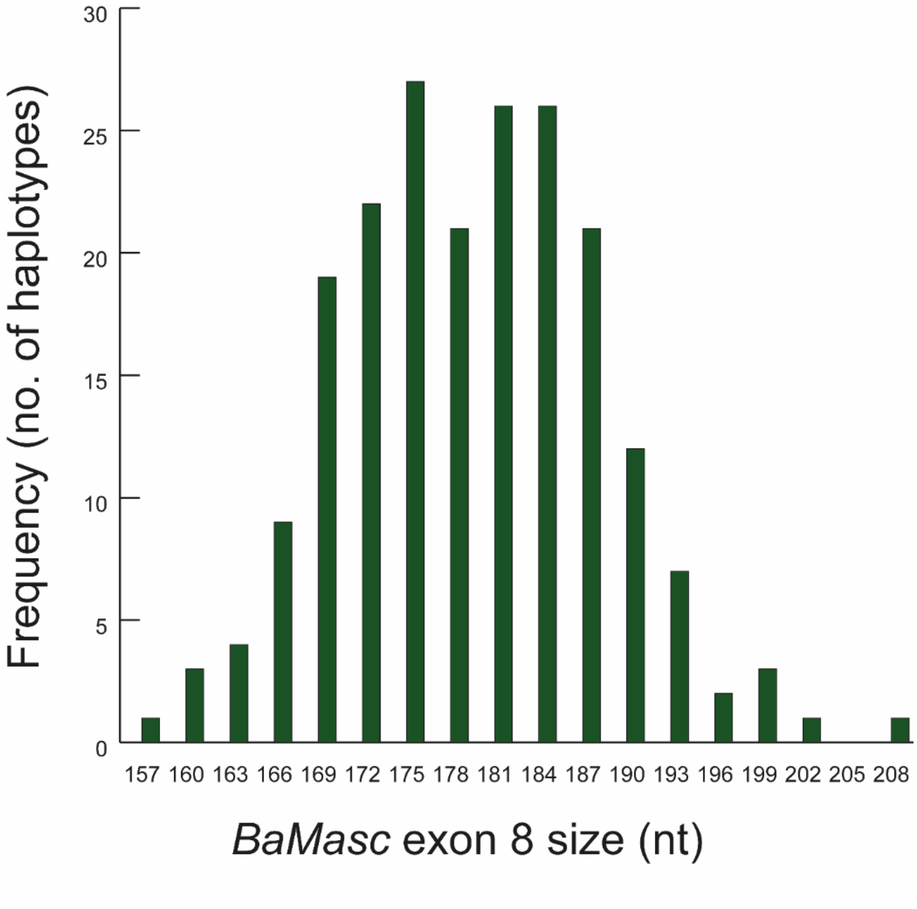

Fig. S9. Length variation of *BaMasc* exon 8

Frequency distribution of the length of *BaMasc* exon 8 (in nucleotides), due to length variation in HVR, in a sample of 246 females (205 distinct *BaMasc* haplotypes).

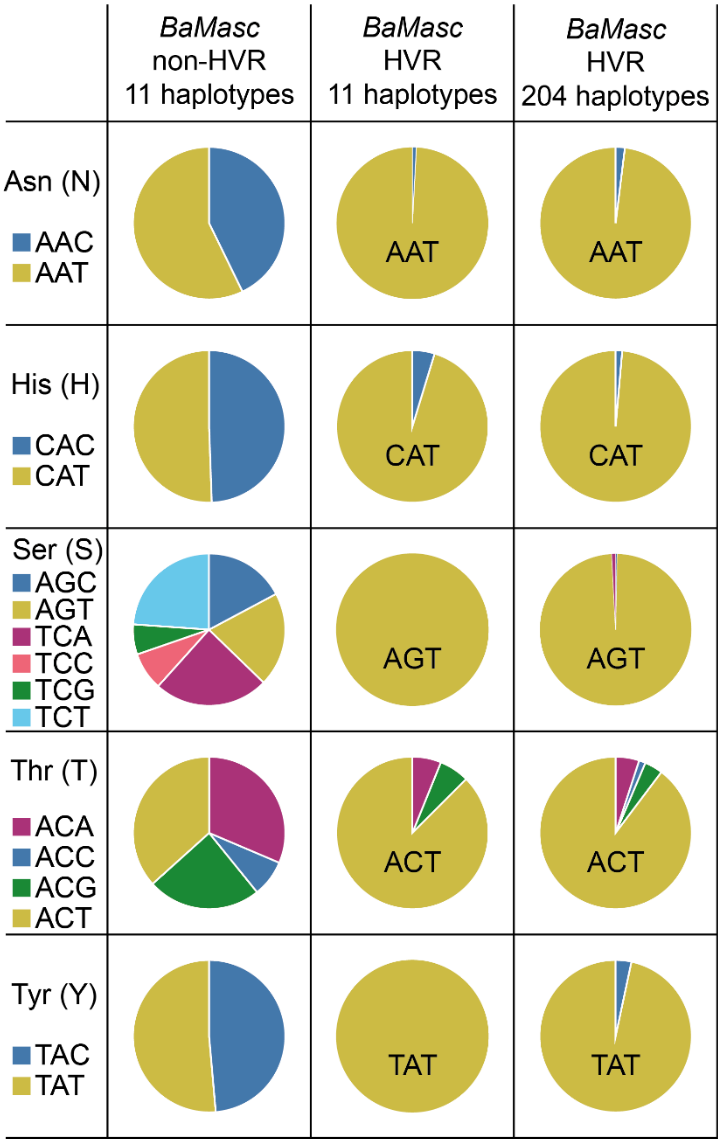

Fig. S10. Biased codon usage in *BaMasc* HVR

BaMasc HVR is almost exclusively composed of five amino acids (asparagine, histidine, serine, threonine, tyrosine), each encoded by 2–6 synonymous triplet codons. The pie charts show the proportional usage of codons (represented by different colors) encoding each of these five amino acids. Codon usage was calculated separately for HVR and the whole of *BaMasc* excluding HVR (non-HVR), based on 11 haplotypes for which complete sequence was available. The same extreme bias in codon usage pattern is observed in a larger sample of 204 *BaMasc* HVR haplotypes.

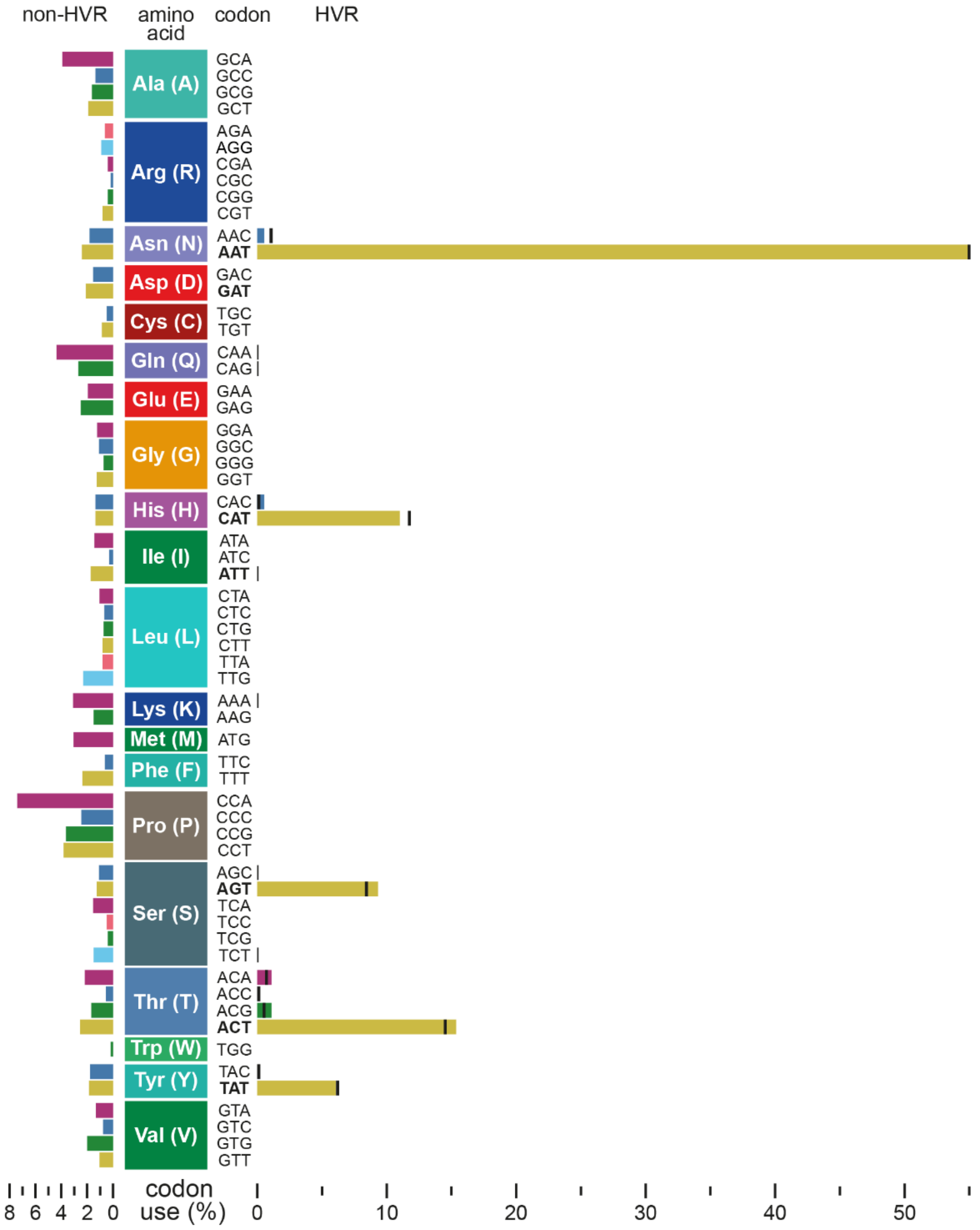

Fig. S11. Restricted amino acid and biased codon usage in *BaMasc* HVR

Colored boxes show the three (and single) letter codes for all 20 amino acids, with their corresponding codons to the right. ANT and NAT codons are highlighted in bold. Horizontal bars represent average percentages of codons present within the HVR (right), contrasted with the rest of *BaMasc* (left), based on 11 complete haplotypes. The black vertical lines indicate the HVR codon percentages based on 204 haplotypes, showing the same overall pattern as the 11 haplotypes. All 61 codons are present in the non-HVR region without notable differences in iso-triplet usage. The HVR is dominated by five ANT/NAT codons which encode five amino acids (N, H, S, T, Y), to the near exclusion of alternative synonymous codons. The remaining two ANT/NAT codons, encoding D and I, are effectively absent from HVR.

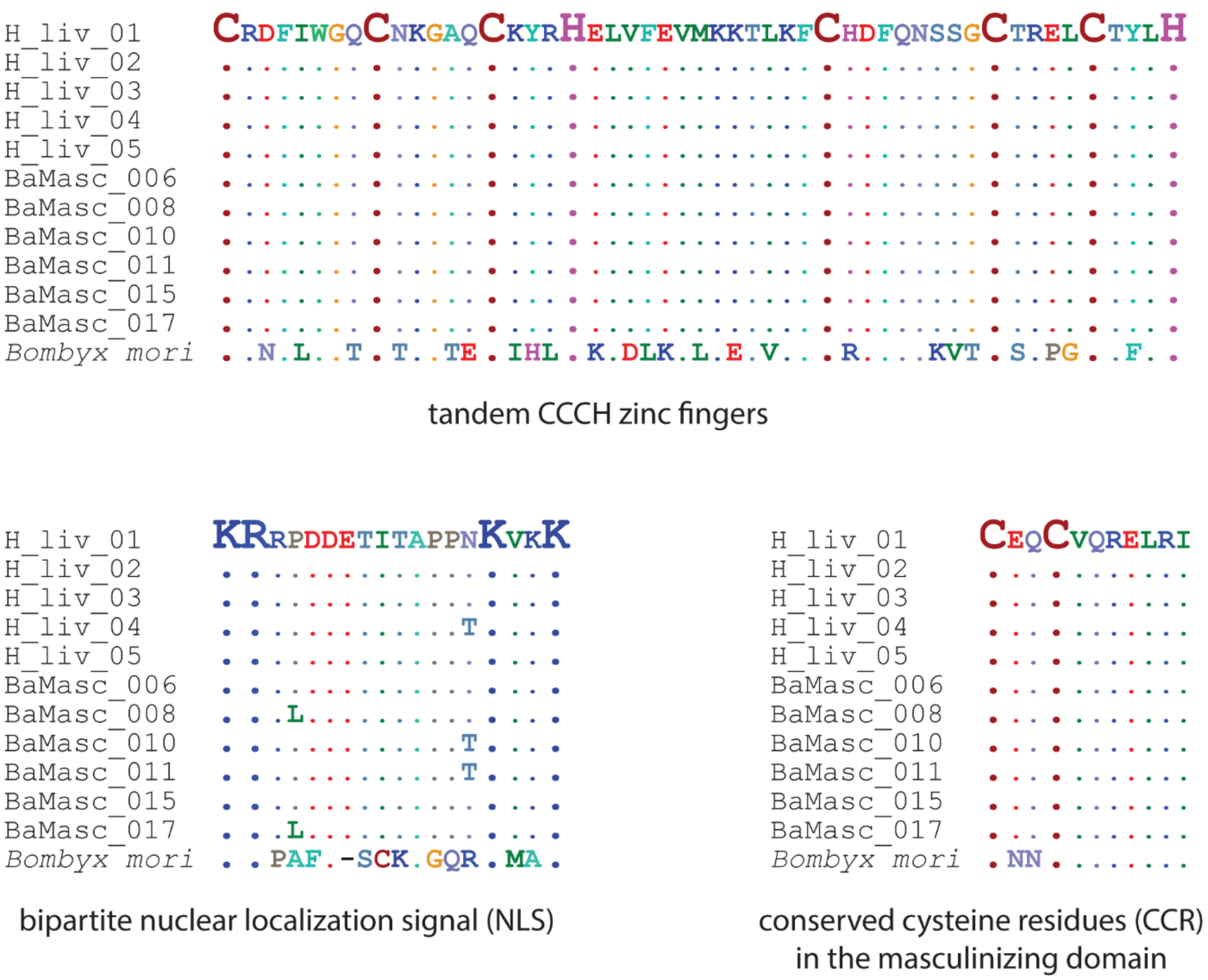

Fig. S12. Structurally conserved domains in *Bicyclus anynana Masc* and their homologs in *Bombyx mori Masc*

Amino acid alignments of the three structurally conserved domains in 11 *BaMasc* haplotypes and the *BmMasc* homologs consisting of tandem CCCH zinc fingers, the bipartite nuclear localization signal (NLS), and the conserved cysteine residues (CCR) in the masculinizing domain. The domain-defining amino acids (CCCH, KRKK, and CC respectively) are shown in larger font. Dots, represent amino acids identical to the top row.

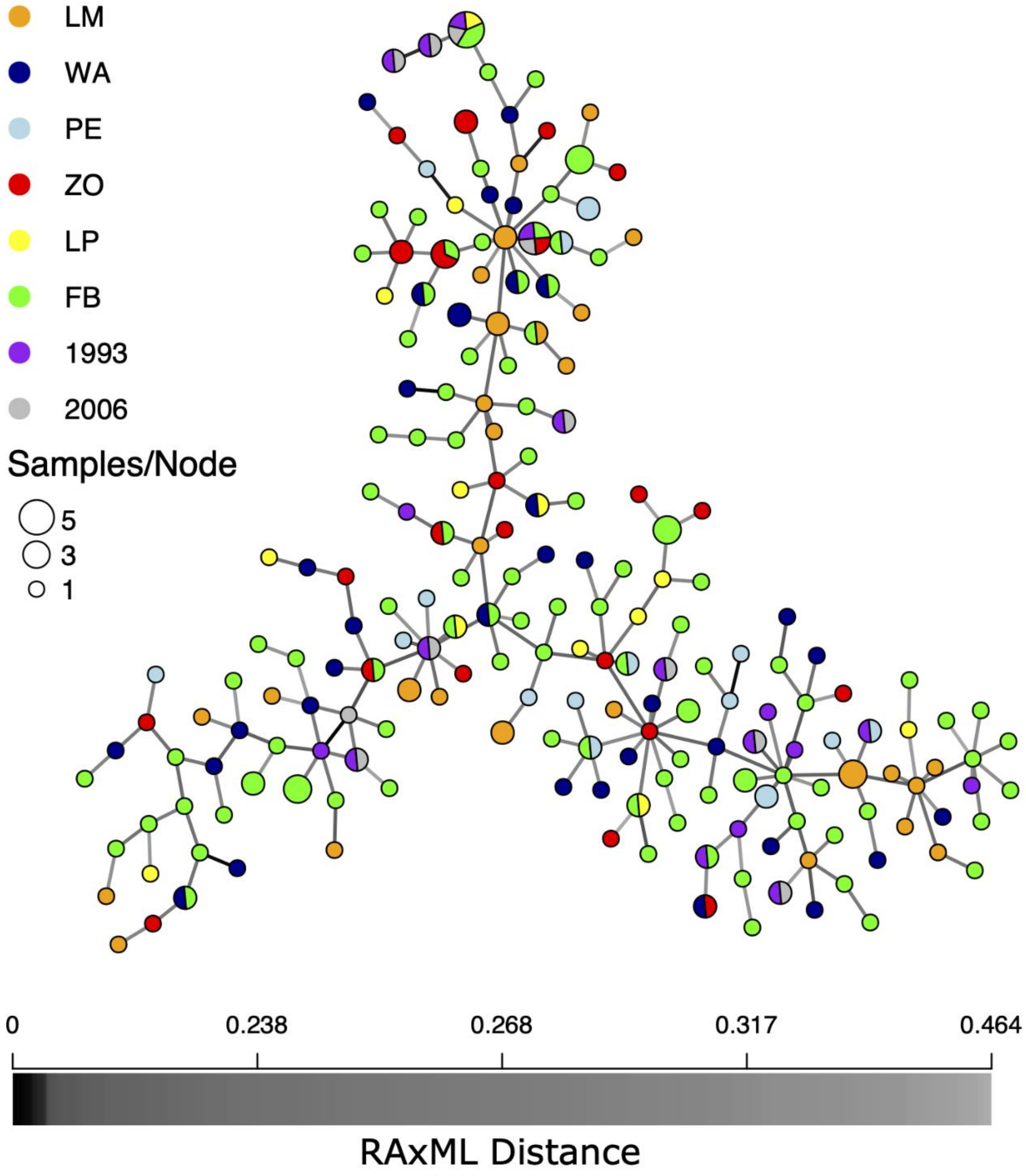

Fig. S13. Haplotype network for *BaMasc* E8–9

BaMasc exons 8+9 aa haplotype network, including the HVR, based on an effective sample of 243 females (including any haplotypes shared between Leiden$_{1993}$ and Liverpool$_{2006}$), containing 204 distinct aa haplotypes. Branch lengths between neighboring nodes are effectively fixed and do not reflect variation in genetic distances among haplotypes, which is instead indicated by the darkness of the lines (RAxML distance scale). Node colors indicate the geographic origin of the haplotypes (LM - Lake Mburo, WA - Watamu, PE - Pemba, ZO - Zomba, LM - Limpopo, FB - False Bay, 1993 - Leiden, 2006 - Liverpool).

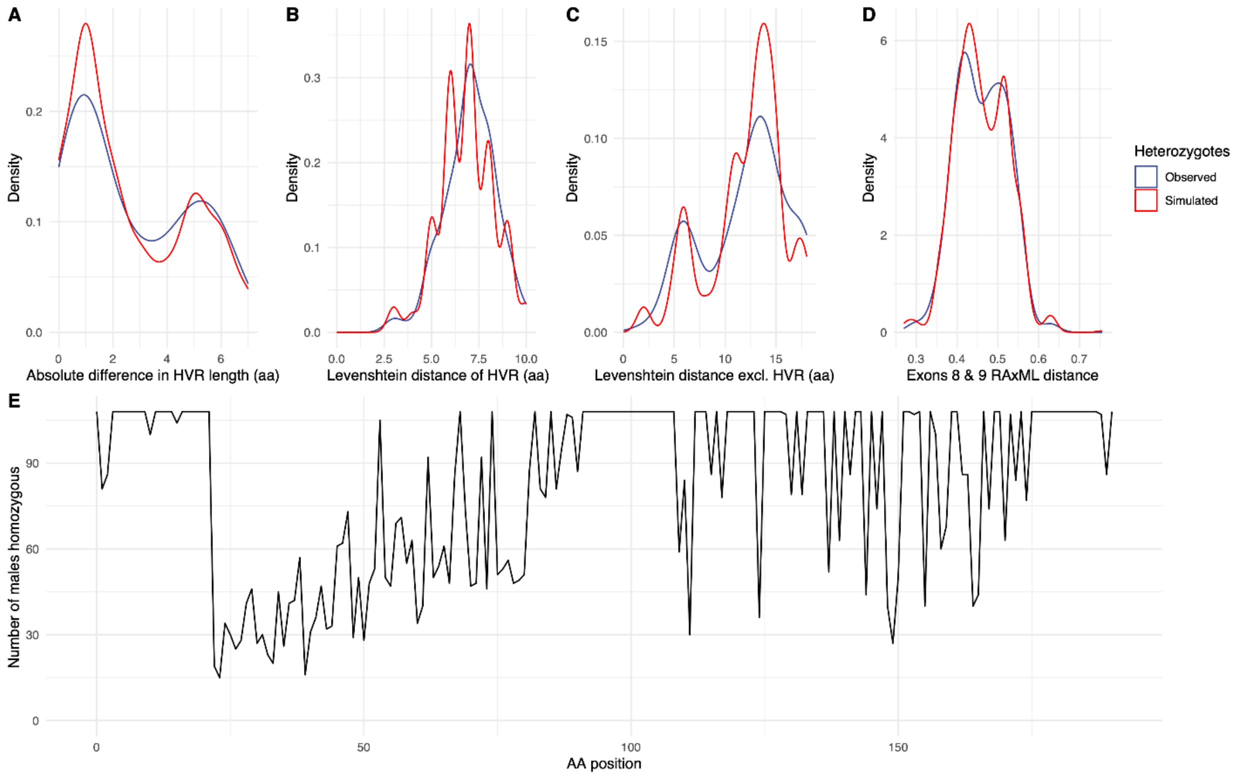

Fig. S14. Distribution of *BaMasc* sequence differences

Observed vs simulated distributions of pairwise genetic distance between *BaMasc* E8–9 haplotype combinations in heterozygous males. The four measures of genetic distance, all relating to aa sequence, are: (**A**) absolute difference in HVR length; (**B**) Levenshtein distance for the unaligned HVR only; (**C**) Levenshtein distance for the aligned exon 8–9 sequence excluding the HVR; (**D**) a combined measure for the full sequence (RAxML distance). The analysis is restricted to the 18 haplotypes observed in the Leiden 1993 male sample. (**E**) The number of males homozygous at each aa position of *BaMasc* E8–9 sequence observed in the Leiden 1993 adult male sample (*n* = 108). For the HVR, which is challenging to align, the PICS-Ord numeric encoding of the aa sequence was used, rather than the aa themselves.

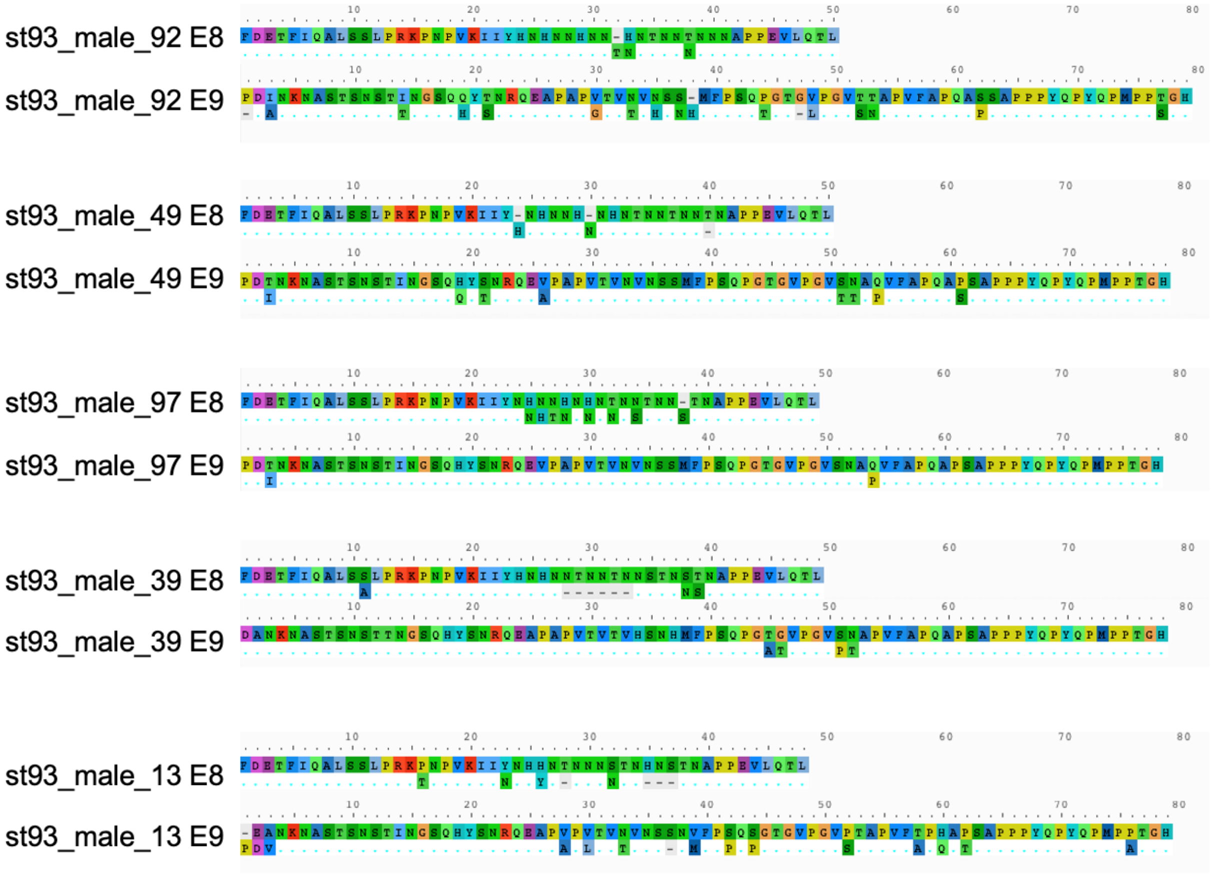

Fig. S15. Minimal and maximal sequence differences in functional *BaMasc* E8–9 heterozygotes

A small selection of aligned functional pairs of *BaMasc* E8–9 aa sequences (observed in Leiden 1993 adult males), which show minimal (upper four males) and maximal (bottom male) divergence from each other, according to different genetic distance criteria. From top to bottom: Levenshtein of HVR only (st93_male_92 and st93_male_49); Levenshtein excluding HVR (st93_male_97); RAxML of composite E8-9 sequence (st93_male_39); RAxML of composite E8-9 sequence (st93_male_13). Intra-male haplotype alignments were performed with MAFFT (v7.205) and manually edited. The sequences of each exon are approximately 80% complete.

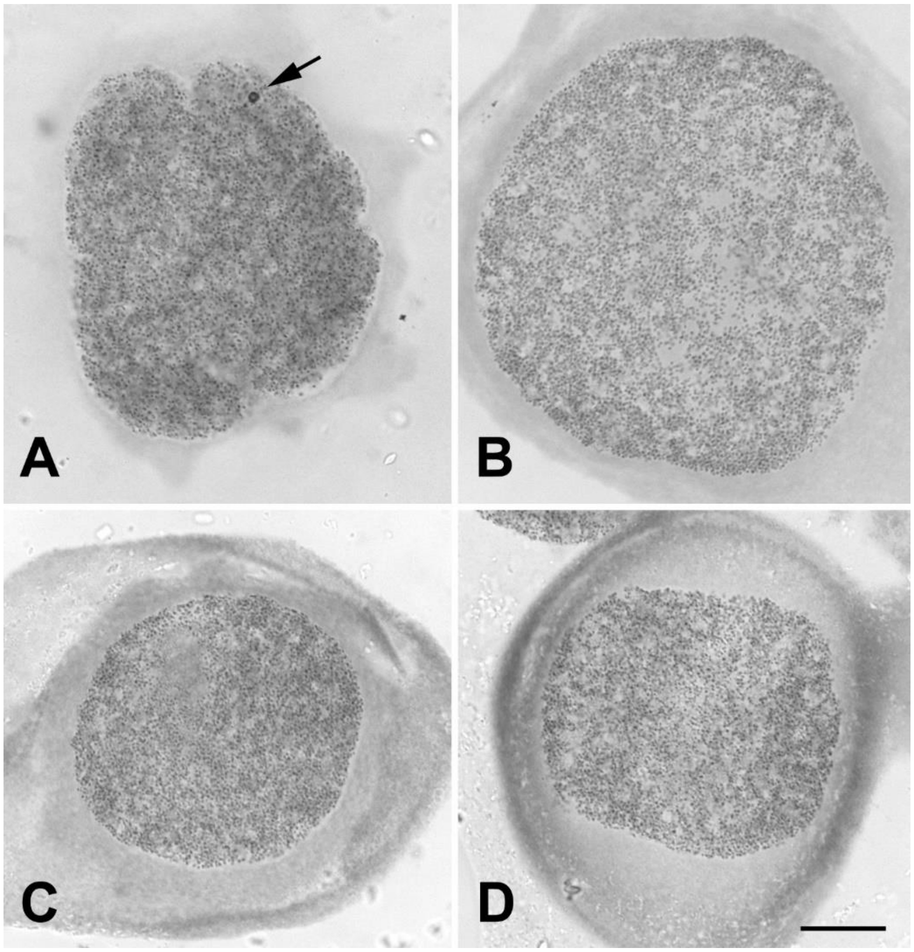

Fig. S16. Evidence for the existence of Z0 females in *Bicyclus anynana* (absence of W chromatin body)

Highly polyploid nuclei of Malpighian tubule cells in *Bicyclus anynana* larvae, stained with lactic acetic orcein. (**A, B**) WZ female line. (**C, D**) Z0 female line. (A) A female nucleus showing a deeply stained W chromatin body (arrow). (B) A male nucleus without W chromatin body. (C) A female nucleus without W chromatin body. (D) A male nucleus without W chromatin body. Bar = 20 μm.

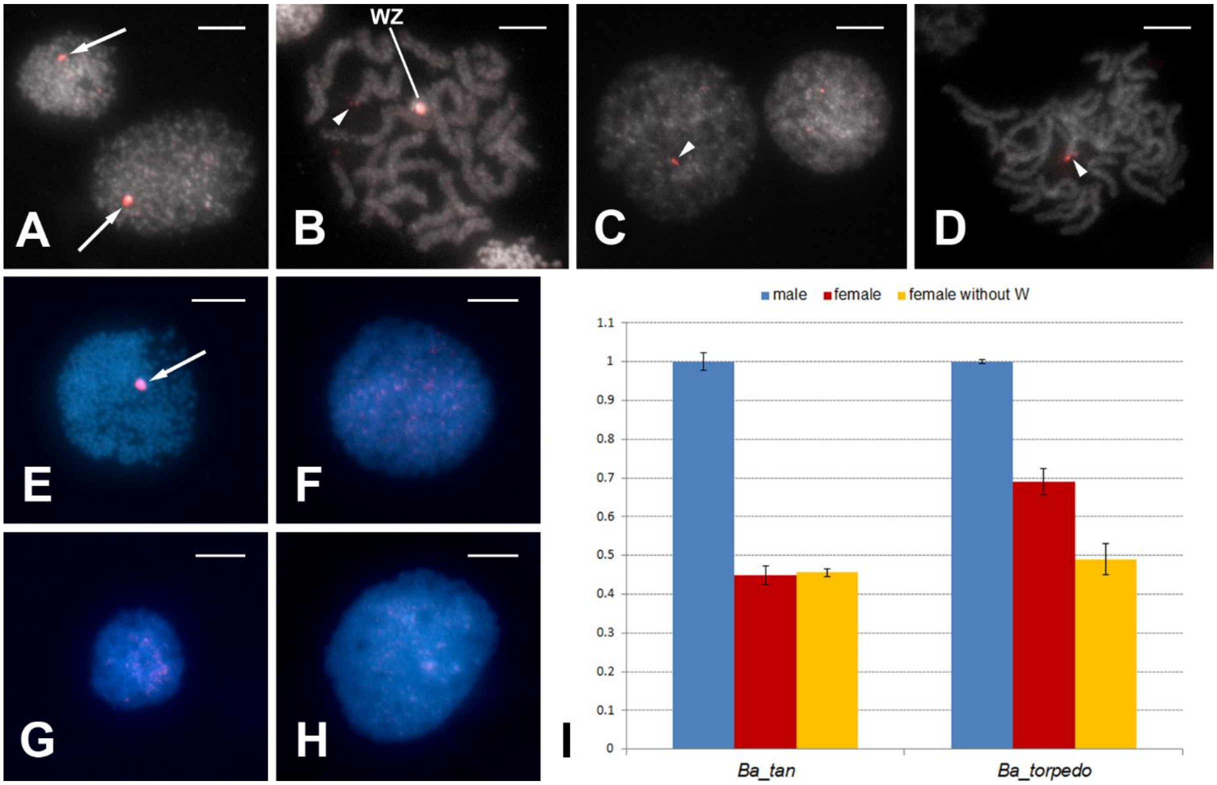

Fig. S17. Evidence for the existence of Z0 females in *Bicyclus anynana* by analysis of frozen pupa remains

(**A**–**H**) Fluorescence in situ hybridization (FISH) with the W chromosome-painting probe (red). Nuclei and chromosomes were counterstained with DAPI (grey in A–D, blue in E–H). Arrows point to sex chromatin formed by the W chromosome. Arrowheads point to additional hybridization signals of the W-painting probe that localize the autosomal cluster of major rDNA. Bar = 10 µm. (A–D) FISH on preparations from gonads of female (A, B) and male (C, D) larvae of the standard laboratory population used as control. (A) Two interphase nuclei, each with a sex chromatin body. (B) Meiotic chromosomes of a pachytene oocyte with a peculiar WZ bivalent in which the Z chromosome strand is wrapped around the body-like W chromosome. (C) Two interphase nuclei without sex chromatin. (D) Meiotic chromosome bivalents of a pachytene spermatocyte. (E–H) FISH on preparations of interphase nuclei from tissues of frozen pupae. (E) A nucleus of a WZ female with a sex chromatin body. (F) A nucleus of a ZZ male without sex chromatin. (G, H) Nuclei of a Z0 female showing no sex chromatin. The smaller nucleus (G) is probably in G_1 phase and the larger nucleus (H) in G_2 phase. (**I**) Quantitative PCR (qPCR) comparison of the relative doses of the Z-linked genes *Ba_tan* and *Ba_torpedo* normalized to the *B. anynana* ortholog of the autosomal reference gene *Aos1* (*Activator of SUMO 1*) on chromosome 19. Genomic DNAs from frozen pupae of a ZZ male, a WZ female, and a Z0 female were used as templates. Error bars represent SDs calculated from three independent samples. In both female samples, the doses of the two Z-linked genes differed significantly from the male sample, i.e. from the 1:1 ratio ($P < 0.05$), but there were no significant differences between the female samples (statistically analyzed by Welch's *t*-test with a Bonferroni correction for multiple testing), thus demonstrating the hemizygosity of the Z chromosome in both WZ and Z0 females.

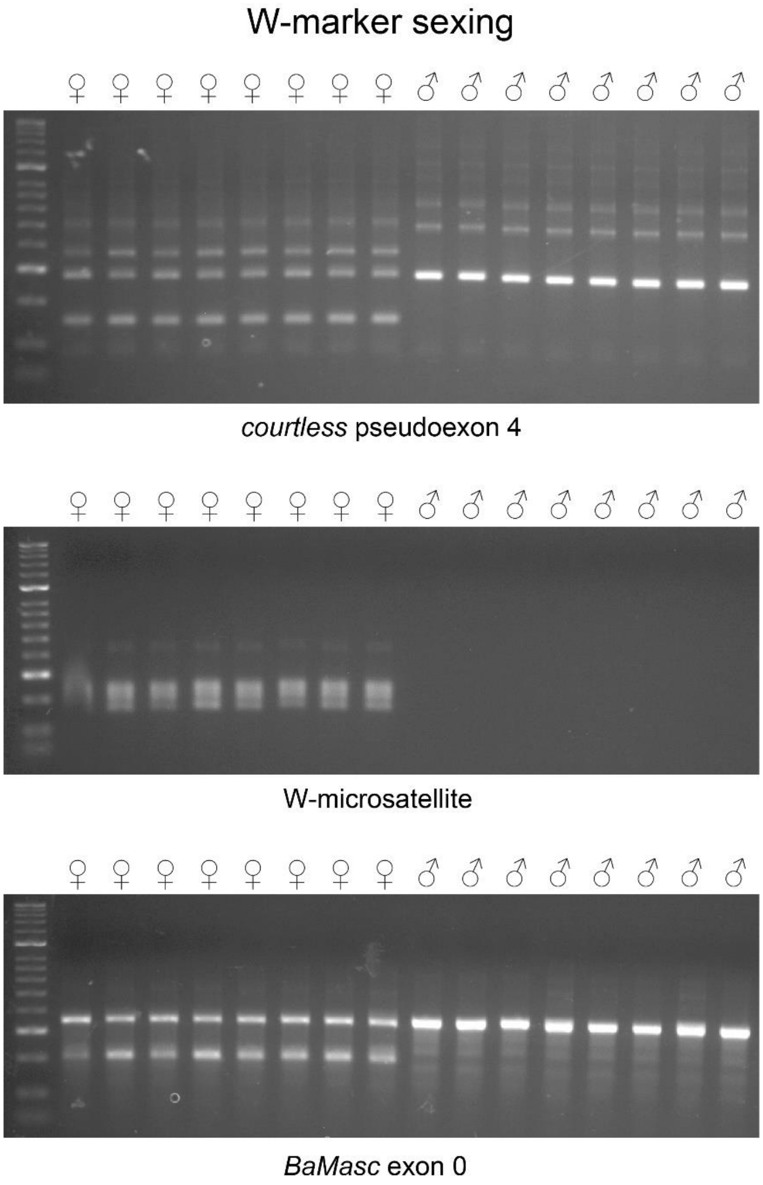

Fig. S18. ***Bicyclus anynana*** **W-chromosome markers gel electrophoresis**

Three W-markers showing sex-specific bands on agarose gels for 16 full-sibs consisting of 8 sisters and 8 brothers. The *courtless* gel shows a banding pattern caused by tandemly repeated non-functional genomic copies of exon 4 (pseudoexons) with two bands occurring exclusively in females. The W-microsatellite is present in all females and absent in all males (the second well had imperfections, resulting in a smudged band). The *BaMasc* exon 0 pattern shows an indel polymorphism with the smaller band representing the W-copy present only in females and the larger band the Z-copy present in both females and males.

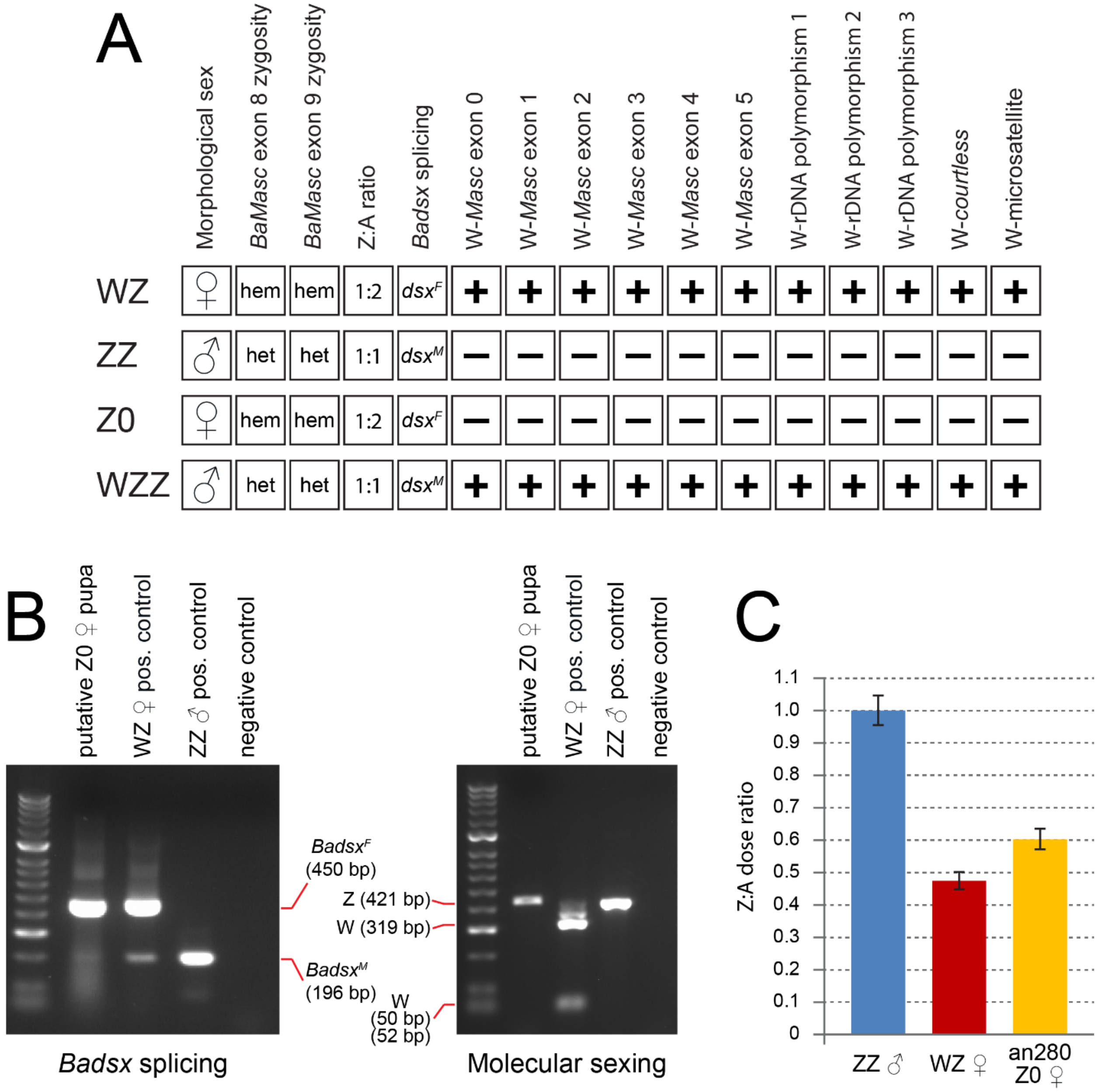

Fig. S19. Validation of putative Z0 females using genetic markers

(**A**) Table of non-cytogenetic criteria used to establish sex chromosome compositions WZ, ZZ, Z0, and WZZ. The first five columns show the characteristics associated with sex, the remaining columns show the presence (+) or absence (–) of 11 W-chromosome markers. (**B**) *Badsx* splicing and molecular sexing assay of the putatively Z0 pupa, against known positive and negative controls. (**C**) Z:A dose ratio for ZZ male, WZ female and a putatively Z0 wild-caught female (an280) from Lake Mburo, Uganda. The mean and standard deviation of each ratio was estimated by genomic qPCR from single individuals in triplicate using one Z-linked gene and one autosomal gene, calibrated relative to the ZZ male dose.

Movie S1.

Timing of the maximum incubation period to hatching of *Bicyclus anynana* embryos. Time lapse series of a sample of developing eggs, collected en masse during a 60 min window from a laboratory stock population. Eggs were incubated at 26°C. The series covers the period from 70–133 hours post oviposition (hpo), +/- 30 min. The majority of larvae hatch between 98 and 103 hpo. No larvae hatch after 108 hpo. Dark larval head capsules begin to appear at 76 hpo. At the end of the series, approximately 12 eggs show the 'black egg' phenotype characteristic of *BaMasc* homozygotes, and 10 show the 'white egg' phenotype.

Table S1.

Lethal interval recombination matrix. Genotypes of 24 brothers (family $2BF_1$) at 37 Z-linked loci selected to define the boundaries of the homozygous lethal interval.

Table S2.

***W-BaMasc* exon copy number.** Estimated copy number and nucleotide sequence for exons 0–5 of *W-BaMasc*. There are no copies of exons 6–10.

Table S3.

Primers. All primer sequences used in this study (excluding those for recombination mapping markers, which are listed in Table S2).

Table S4.

Viability of *BaMasc* homozygotes. *BaMasc* genotype, karyotypic sex and viability phenotype of 21 broods produced by full-sib crosses.

Table S5.

***BaMasc* haplotype frequencies in laboratory and wild samples.** *BaMasc* genotype, (and/or zygosity), and karyotypic sex in adult female and male samples of *Bicyclus anynana* from two laboratory and six natural populations. The file also includes the frequency of each haplotype and the haplotype frequency distribution.

Table S6.

***Badsx* splice isoforms in relation to *BaMasc* zygosity.** *BaMasc* genotype, karyotypic sex, and *Badsx* isoform of *Bicyclus anynana* embryos at 100 hpo and 69 hpo.

Table S7.

Data for *BaMasc* siRNA knockdown experiment. Sex karyotype, *Badsx* isoform, and *BaMasc* zygosity for a sample of *Bicyclus anynana* embryos subjected to one of three siRNA treatments (negative control, Dsi-01 or Dsi-02).

Table S8.

***BaMasc* exon 8 and 9 haplotype sequences.** Listing (identifiers and nucleotide sequences) of all the *BaMasc* exon 8 and 9 haplotypes detected in this study. Also listing the nucleotide sequence identifiers used in the alignment figure comparing HVR in *Bicyclus anynana Masc* and *Apis mellifera csd*.
